# Effective gene therapy for haemophilic mice with pathogenic factor IX antibodies

**DOI:** 10.1002/emmm.201302859

**Published:** 2013-09-16

**Authors:** David M Markusic, Brad E Hoffman, George Q Perrin, Sushrusha Nayak, Xiaomei Wang, Paul A LoDuca, Katherine A High, Roland W Herzog

**Affiliations:** 1 Department of Pediatrics, University of FloridaGainesville, FL, USA; 2 Department of Pediatrics, The Children's Hospital of Philadelphia and University of Pennsylvania Medical CenterPhiladelphia, PA, USA; 3 Center for Cellular and Molecular Therapeutics, The Children's Hospital of PhiladelphiaPhiladelphia, PA, USA; 4 Howard Hughes Medical InstitutePhiladelphia, PA, USA

**Keywords:** adeno-associated virus, factor IX, haemophilia B, inhibitors, liver gene transfer

## Abstract

Formation of pathogenic antibodies is a major problem in replacement therapies for inherited protein deficiencies. For example, antibodies to coagulation factors (‘inhibitors’) seriously complicate treatment of haemophilia. While immune tolerance induction (ITI) protocols have been developed, inhibitors against factor IX (FIX) are difficult to eradicate due to anaphylactic reactions and nephrotic syndrome and thus substantially elevate risks for morbidity and mortality. However, hepatic gene transfer with an adeno-associated virus (AAV) serotype 8 vector expressing FIX (at levels of ≥4% of normal) rapidly reversed pre-existing high-titre inhibitors in haemophilia B mice, eliminated antibody production by B cells, desensitized from anaphylaxis (even if protein therapy was resumed) and provided long-term correction. High levels of FIX protein suppressed memory B cells and increased Treg induction, indicating direct and indirect mechanisms of suppression of inhibitor formation. Persistent presence of Treg was required to prevent relapse of antibodies. Together, these data suggest that hepatic gene transfer-based ITI provides a safe and effective alternative to eradicate inhibitors. This strategy may be broadly applicable to reversal of antibodies in different genetic diseases.

→See accompanying article http://dx.doi.org/10.1002/emmm.201302857

## INTRODUCTION

Treatment of inherited protein deficiency may be complicated by pathogenic antibody responses during replacement therapy, highlighting the need for development of suitable immune tolerance protocols. One example is haemophilia B, which results from the loss of functional coagulation factor IX (FIX) with an estimated incidence of 1 in 30,000 male births worldwide. Untreated, patients can develop spontaneous bleeds into the joints and closed spaces resulting in high morbidity and mortality. Disease severity is based on residual FIX activity (severe <1%, moderate 1–5% and mild >5%), which impacts the frequency and severity of bleeds. Patients are currently treated with exogenous FIX protein concentrate, which is plasma-derived or recombinant. A fraction of patients (2–5%), predominantly those with severe haemophilia B, develop neutralizing antibodies to the FIX, termed inhibitors, requiring treatment with expensive bypassing agents to maintain haemostasis. Most of the available bypassing products are designated for short-term treatment on-demand use, and thus haemophilia B patients with inhibitors experience increased morbidity. Unfortunately, haemophilia B patients have a poor response rate to immune tolerance induction (ITI) protocols that require frequent high levels of factor administration. ITI often has to be stopped because of anaphylaxis or nephrotic syndrome (Chitlur et al, 2009; DiMichele, 2007; DiMichele, 2012; Ewenstein et al, 1997; Jadhav & Warrier, 2000; Recht et al, 2011). IgE formation has been identified as a cause for anaphylactic reactions against FIX, which occur in 25–50% of inhibitor patients (Jadhav & Warrier, 2000; Recht et al, 2011; Thorland et al, 1999; Warrier et al, 1997). Because of the severity of the immune response and lack of effective tolerance protocols, inhibitor formation in haemophilia B has been described as an ‘orphan disease in need of attention’ (DiMichele, 2007).

Toward the goal of preventing inhibitor formation in haemophilia B, we demonstrated that hepatic adeno-associated viral (AAV) gene transfer induces FIX-specific immune tolerance (Cao et al, 2007; Dobrzynski et al, 2006; Mingozzi et al, 2003). This *in vivo* gene transfer approach is very attractive since it simultaneously provides therapy and immune tolerance, and the concept has since been adapted to multiple other inherited protein deficiencies, including lysosomal storage disorders (Koeberl & Kishnani, 2009; LoDuca et al, 2009). For treatment of haemophilia B, AAV liver gene transfer has been successful in small (Cooper et al, 2009; Dobrzynski et al, 2006; Markusic et al, 2010; Mingozzi et al, 2003) and large animal models (Niemeyer et al, 2009) and, most recently, in human clinical trial (Manno et al, 2006; Nathwani et al, 2011). Sustained FIX expression at levels of ∼6% of normal has now been achieved in several subjects (Davidoff et al, 2012). In two different liver directed AAV-*hF9* gene transfer clinical trials there has been no indication of B- or T-cell responses directed against FIX (Manno et al, 2006; Nathwani et al, 2011). However, CD8^+^ T-cell responses against viral input capsid have limited levels and/or duration of expression in some subjects, a problem that was solved by transient immune suppression with the steroid drug prednisolone and that can be further minimized by use of capsid sequences engineered to reduce MHC I presentation (Markusic et al, 2010; Martino et al, 2013; Zhong et al, 2008).

TGF-β-dependent induction of regulatory CD4^+^CD25^+^FoxP3^+^ T cells (Treg) is a critical component of the mechanism of tolerance induction by hepatic AAV gene transfer (Hoffman et al, 2011; Cao et al, 2007; Dobrzynski et al, 2004, 2006). Induced Treg actively suppress antibody and T-cell responses against FIX. Tolerance induction has been further improved by use of AAV serotype 8 vector or mutant AAV2 devoid of several surface-exposed tyrosine residues, thereby reducing proteasomal processing following cellular entry (Cooper et al, 2009; Markusic et al, 2010). With these modifications, we were able to achieve immune tolerance in haemophilia B mice on a genetic background that predisposes to elevated immune responses against FIX (Cooper et al, 2009; Markusic et al, 2010). Moving forward it will be important to determine the safety of AAV liver gene transfer in inhibitor patients or patients with a previous history of inhibitors. However, we had been unable to ask the logical question of whether this protocol could be an alternative to current clinical ITI and safely and effectively reverse inhibitors to FIX until recently, when we developed an animal model for anaphylaxis in FIX replacement therapy. C3H/HeJ mice with a gene deletion for murine *F9* (C3H/HeJ *F9*^−/−^) develop high-titre inhibitors and fatal anaphylaxis following weekly intravenous (IV) therapy with recombinant human FIX through a combination of IgG1 and IgE formation (Verma et al, 2010). Using this and other models, we show in the following that (i) risk of FIX inhibitor formation in this haemophilic mouse strain resembles the human experience; (ii) hepatic AAV gene transfer safely and effectively reverses inhibitors by shutting down antibody production by B cells while also desensitizing from anaphylaxis, resulting in sustained therapy and (iii) sustained Treg-mediated suppression is required.

## RESULTS

### Murine haemophilia B model of anaphylaxis in FIX replacement therapy

**Figure 1:**
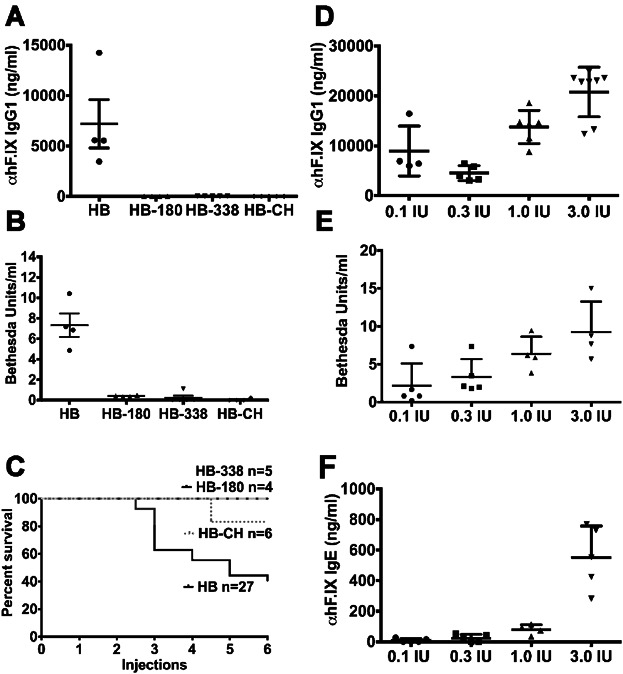
Mutation and dose dependence of antibody responses against hFIX in hemophilia B mice C3H/HeJ *F9*^−/−^ mice (HB) and C3H/HeJ *F9*^−/−^ mice transgenic for mutant hFIX proteins R180W (HB-180), LS 338 (HB-338), and G381E (HB-CH) were challenged with 1 IU hFIX protein starting with an intraperitoneal injection following by 5 weekly intravenous injections. Anti-hFIX IgG1 titers (ng/ml) one week following the last hFIX protein injection.Inhibitor (Bethesda) titers one week following the last hFIX protein injection.Kaplan-Meier survival plot for HB, HB-180, HB-338, HB-CH mice following weekly challenge with hFIX protein. HB mice were co-administered antihistamine and PAF antagonist on the fifth and sixth exposures to hFIX protein.HB mice were immunized with a range of hFIX protein levels (0.1 to 3 IU, which corresponds to 0.5 to 15 µg total hFIX protein per dose). Shown are anti-hFIX IgG1 titers (ng/ml) at the end of challenge as a function of hF.IX dose.Bethesda titers (BU/ml) as a function of hF.IX dose in HB mice.Anti-hFIX IgE levels (ng/ml) as a function of hF.IX dose in HB mice. Graphs in A, B, D-F show data for individual animals as well as averages ± SD; *n* = 4–6/experimental group.

We have previously described a murine haemophilia B model, C3H/HeJ background with a *F9* gene deletion (C3H/HeJ *F9*^−/−^), that develops both anti human factor IX (hFIX) IgG1 and IgE antibodies following weekly IV administration of hFIX (Markusic et al, 2010; Nayak et al, 2009; Verma et al, 2010). These mice develop fatal anaphylactic reactions to hFIX protein following repeated exposures with a loss of ∼50% of the challenged mice by the fifth exposure (Fig 1C). These responses are specific to the C3H/HeJ background as BALB/c *F9*^−/−^ mice, with the same gene deletion, tolerated up to eight weekly challenges of hFIX protein with only mild IgG1 responses (Lozier et al, 2005) and absence of fatal anaphylaxis (unpublished observations). Clinically, the risk of developing inhibitors or anaphylaxis in haemophilia B patients is greater in patients with gene deletion or complex chromosomal rearrangements, followed by nonsense mutations, and then missense mutations (Chitlur et al, 2009). We maintain three different C3H/HeJ *F9*^−/−^ lines transgenic for hFIX mutants, two of which are cross reactive immunological material negative (crim−) (late stop codon AA 338 and a G381E missense mutation analogous to a spontaneous mutation in the canine haemophilia B colony at Chapel Hill) and a crim+ mutant (R180W), referred to as HB-338, HB-CH and HB-180, respectively. In contrast to control C3H/HeJ *F9*^−/−^ mice, all three transgenic mutant hFIX mice groups tolerated weekly hFIX challenge with no evidence for antibody/inhibitor formation against hFIX IgG1 (Fig 1A and B). Cumulatively, only 1 of 15 mice with endogenous hFIX expression died during the course of the experiment, and no signs of anaphylaxis were observed, while only 40% of gene deletion mice survived (Fig 1C), which showed anaphylactic reactions as previously published (Verma et al, 2010). Thus, the model mirrors the human situation in that endogenous FIX expression confers a level of tolerance that is protective of pathogenic antibody responses.

Although haemophilia A patients treated with factor VIII (F.VIII) protein have a higher incidence of inhibitors (20–30%), remarkably there are few reports of patients with anaphylaxis (Jadhav & Warrier, 2000). One hypothesis is that the higher hFIX systemic levels of total protein needed for therapeutic effect may act as trigger (1 IU hFIX is 5000 ng/ml compared to 200 ng/ml for hF.VIII) (Warrier et al, 1997). Therefore, we evaluated IgE and IgG1 levels in response to weekly challenge with 0.1, 0.3, 1 and 3 IU/ml of hFIX protein (with the two higher doses being in the range clinically used to treat moderate to severe bleeds). We observed a trend towards higher IgG1 and Bethesda inhibitors titres with increasing hFIX protein (Fig 1D and E), while IgE levels strongly correlated with hFIX levels (Fig 1F). Importantly, mice challenged with 0.1 and 0.3 IU hFIX did not require pharmacological intervention to survive the challenge.

### AAV8-*hF9* liver gene transfer reverses hFIX inhibitors, prevents amnestic immune responses including anaphylaxis

Next, we asked if C3H/HeJ *F9*^−/−^ mice with high-titre pre-existing inhibitors could be safely treated with liver gene transfer (or experience anaphylaxis as with continued protein injections) and what would happen to inhibitor levels? We chose an AAV8 vector because it has strong tropism to the liver after peripheral vein administration, avoiding the need for an invasive procedure in the presence of an inhibitor, and fails to transduce professional antigen presenting cells (APCs). Additionally, the construct includes a strong and highly hepatocyte-specific promoter (see published data and Supporting Information Fig S1) (Cooper et al, 2009; Lu & Song, 2009; Manno et al, 2006; Vandendriessche et al, 2007). In naïve animals, efficient hepatic AAV gene transfer can establish a dominant state of immune tolerance that suppresses immune responses to the transgene product in other tissues (Hoffman et al, 2007; Passini et al, 2007; Zhang et al, 2012).

**Figure 2:**
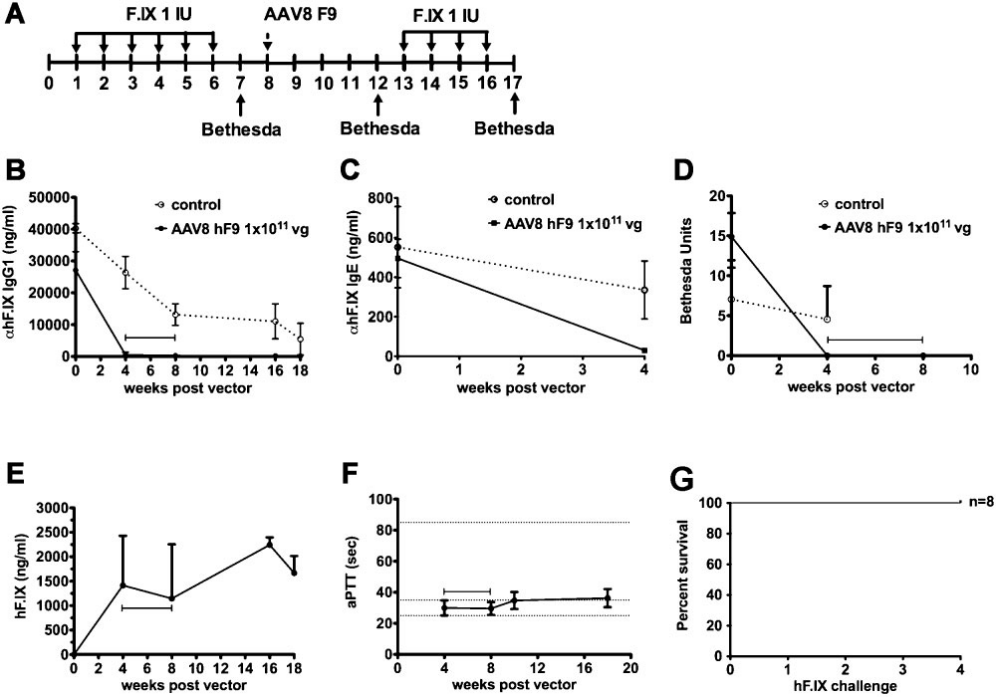
Reversal of inhibitors and of anaphylaxis by hepatic AAV8-*hF9* gene transfer C3H/HeJ *F9*^−/−^ mice (HB) were immunized with hFIX protein to generate pre-existing high-titer inhibitors. A control group of immunized mice were then left alone to follow hFIX antibodies over the course of the experiment. Following confirmation of inhibitors, mice were treated with 1 × 10^11^ vg of an AAV8-*hF9* vector (*n* = 8) and were bled 4, 8, 16, and 18 weeks following injection of vector. Samples from immunized control HB (*n* = 5) and vector-treated HB mice were used for ELISA measurements. Experimental time line.Anti-hFIXIgG1 titers (ng/ml).IgE titers (ng/ml).Inhibitor titers (BU/ml).Circulating levels of hFIX protein (ng/ml).Restoration of hemostasis was assessed by measurement of coagulation activity by activated partial thromboplastin time (aPTT, in seconds). Data points in A–F are average ± SD.Kaplan-Meier survival plot of 1 × 10^11^ vg AAV8-*hF9* vector treated mice following weekly challenge with hFIX protein.

In the first set of experiments, we established a group of mice that had developed high-titre inhibitors (average anti-hFIX IgG1 level of 27 μg/ml, IgE of 330 ng/ml, and Bethesda titre of 16, *n* = 8) after protein therapy (Fig 2A–D). A dose of 1 × 10^11^ vg AAV8-*hF9* vector was delivered via the tail vein 1 week after the last hFIX protein injection. Previous studies conducted in naïve C3H/HeJ *F9*^−/−^ mice showed significant correction with >20% normal hFIX levels using a similar vector dose and delivery route (Cooper et al, 2009). One month following gene transfer mice were bled and challenged for an additional month with weekly IV recombinant hFIX protein in the absence of anti-histamine/PAF antagonist (Fig 2A). Remarkably, 1 month following gene transfer anti-hFIX titres declined to undetectable by IgE and IgG ELISA and Bethesda assays in all vector treated animals (Fig 2B–D). No fatalities or anaphylactic reactions were observed after vector treatment or subsequent hFIX protein injections (Fig 2G). Vector treated mice showed stable hFIX protein expression and complete correction of the aPTT coagulation time through the duration of the experiment (up to 20 weeks post-gene transfer, Fig 2E and F). In the absence of gene transfer and further exposure to hFIX protein, anti-hFIX and Bethesda titres decline much more slowly (Fig 2B and D).

### AAV8-*hF9* liver gene transfer reverses cellular responses to hFIX

**Figure 3:**
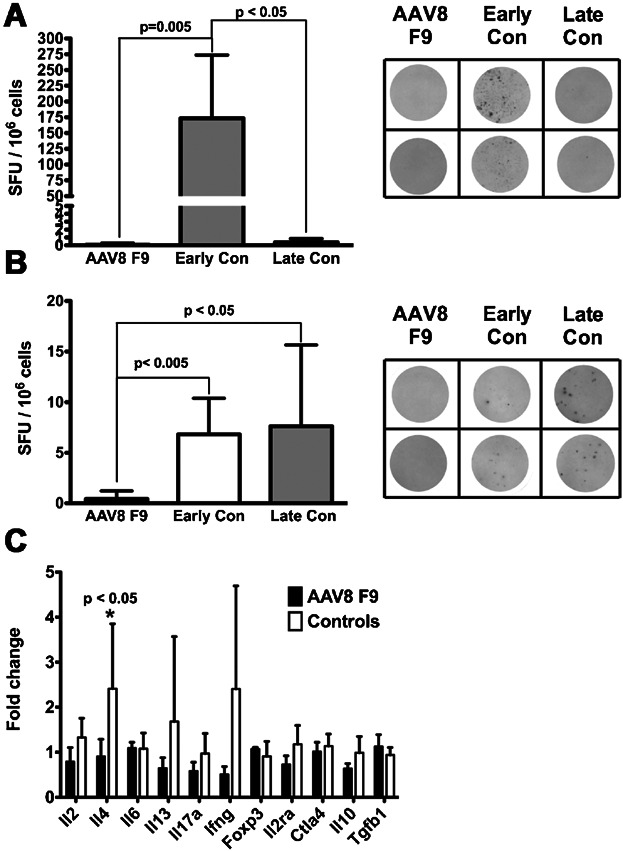
Reversal of B- and T-cell responses against hFIX by hepatic AAV8-*hF9* gene transfer B-cell ELISpot assays specific for hFIX antibody secreting were performed on both bone marrow and spleen cells from C3H/HeJ *F9*^−/−^ mice immunized against hF.X. Results are presented as spots forming units (SFU) per 10^6^ cells with representative wells presented to the right. B-cell ELISpots for hFIX antibody secreting bone marrow cells (AAV8 *F9 versus* Early Con *p* = 0.0022 and AAV8 *F9 versus* Late Con *p* = 0.0286).B-cell ELISpots for hFIX antibody secreting splenocytes (AAV8 *F9 versus* Early Con *p* = 0.0043 and AAV8 *F9 versus* Late Con *p* = 0.0195). Early con (early controls) refers to mice sacrificed shortly after the immunization protocol, *i.e*. the time point of vector treatment. Late Con (late controls) are immunized untreated mice that were followed in parallel to vector treated mice.Cytokine responses specific to hF.IX. Splenocytes (1 × 10^5^ per well) were cultured *in vitro* without or with hFIX protein (10 μg/ml) and harvested 48 h later for mRNA extraction and cDNA synthesis and ran on qPCR array with genes associated with Th1, Th2, or Treg responses. Results are presented as fold change compared to unstimulated cells. Bar graphs indicate average ± SD with *n* = 4/experimental group (IL-4 Control *versus* AAV8 *F9 p* = 0.0196). The students 2-tailed T test was used to determine statistical significance.

Vector treated animals were given a final challenge with hFIX protein and sacrificed the following week to characterize differences in B- and T-cell responses against hFIX protein. Bone marrow and splenocyte cells were harvested for the detection of anti-hFIX secreting cells (ACS) using a B-cell ELISpot. Vector treated mice had no detectable spots (Fig 3A and B), in line with no detectable circulating antibodies for over 3 months (Fig 2B). In contrast, we detected ACS in splenocytes of control mice that had formed inhibitors after protein therapy but did not receive gene transfer (Fig 3A and B). Additional control mice, which were analyzed early after protein therapy (*i.e*. closer to the time when gene transfer was given), showed ACS in spleen at similar frequencies and, additionally, in bone marrow (Fig 3A). Therefore, anti-hFIX producing plasma cells form in the spleen and bone marrow in this model but are persistent in the spleen, and hepatic expression eliminated anti-hFIX production.

Upon *in vitro* stimulation with hFIX antigen, no evidence for a T-cell response in vector treated mice was obtained using quantitative RT-PCR array that included cytokines and markers for Th1, Th2, Th17 and Treg. Control mice (no vector treatment) showed IL-4 expression and additional, albeit with more variability, IL-13 and IFN-γ (Fig 3C). IL-4 and IL-13 are Th2 cytokines known to promote IgG1 and IgE class switch in B cells.

### Effectiveness of inhibitor reversal requires a minimal vector dose

**Figure 4:**
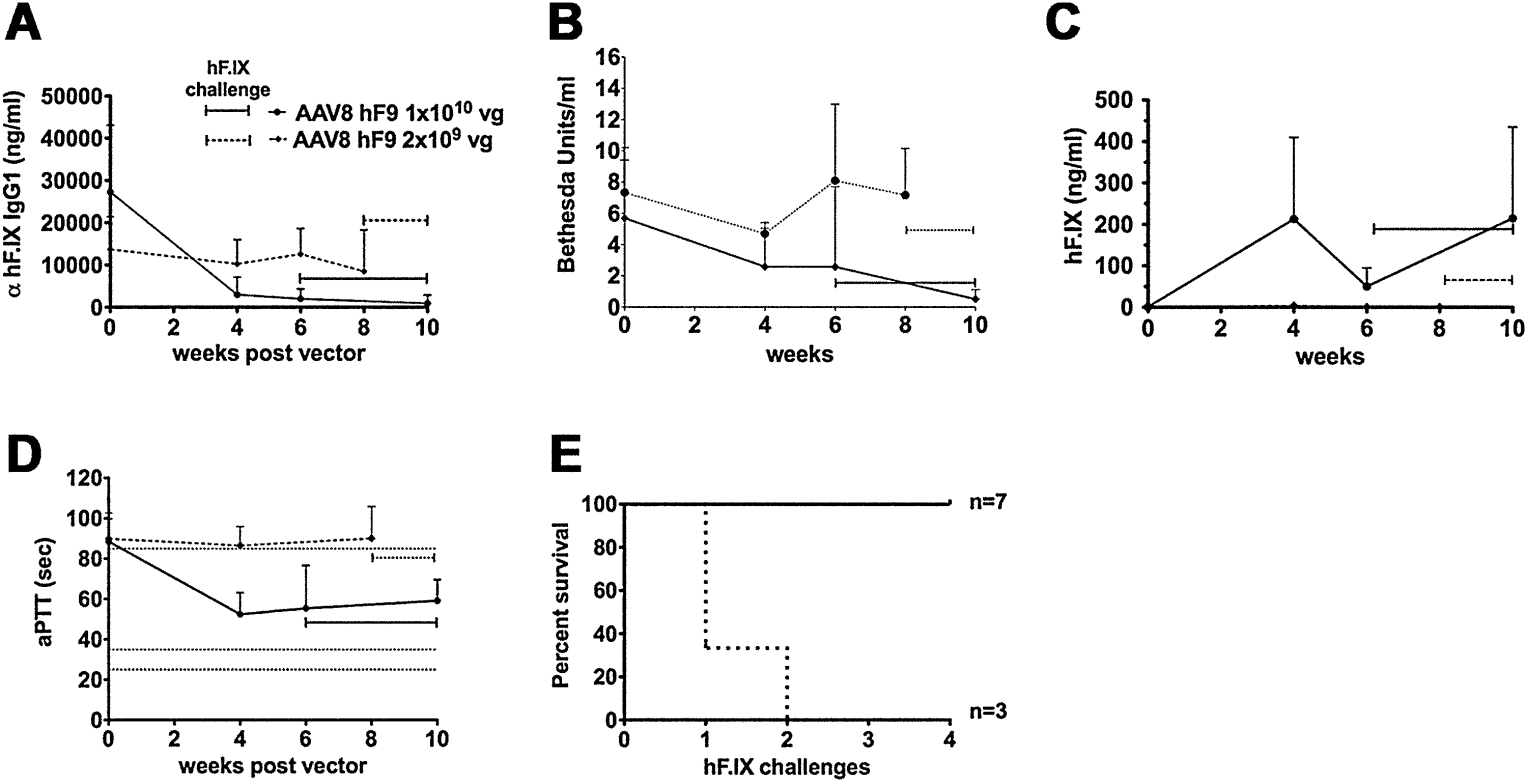
Reversal of B- and T-cell responses against hFIX by hepatic gene transfer is vector dose-dependent Hemophilia B mice were immunized with hFIX protein and subsequently given an IV injection of 1 × 10^10^ (*n* = 7) or 2 × 10^9^ vg (*n* = 3) of AAV8-*hF9* vector. Anti-hFIX IgG1 titers (ng/ml).Inhibitor titers (BU/ml).Circulating hFIX protein levels (ng/ml).Coagulation times (aPTT in sec) as a function of time after vector treatment.Kaplan-Meier survival curves were generated for 2 × 10^9^ and 1 × 10^10^ vg treated HB mice following weekly challenge with hFIX protein. Data points in A-D are average ± SD.

Based on our prior experience with prevention of inhibitor formation, one would predict a minimal expression level to be required for reversal. Therefore, additional mice with inhibitors were treated with an intermediate dose of 1 × 10^10^ or a low dose 2 × 10^9^ vg/mouse. Mice treated with the intermediate dose (*n* = 7) showed a gradual loss in inhibitory antibodies, with a slower kinetics than those treated at the higher dose, while inhibitor titres did not change in the low dose group (*n* = 3, Fig 2D compared to Fig 4B). Neither dose group showed anaphylaxis following gene transfer. A challenge with hFIX protein was again performed, starting 6 and 8 weeks for the 1 × 10^10^ and 2 × 10^9^ vg treated mice, respectively. All mice in the intermediate vector dose group tolerated the challenge with no rise in inhibitors and no indication of anaphylaxis. In contrast, animals in the low dose treated group did not survive the challenge indicating that a dose of 2 × 10^9^ vg was not sufficient to desensitize.

### High hFIX antigen levels suppress memory B cells

**Figure 5:**
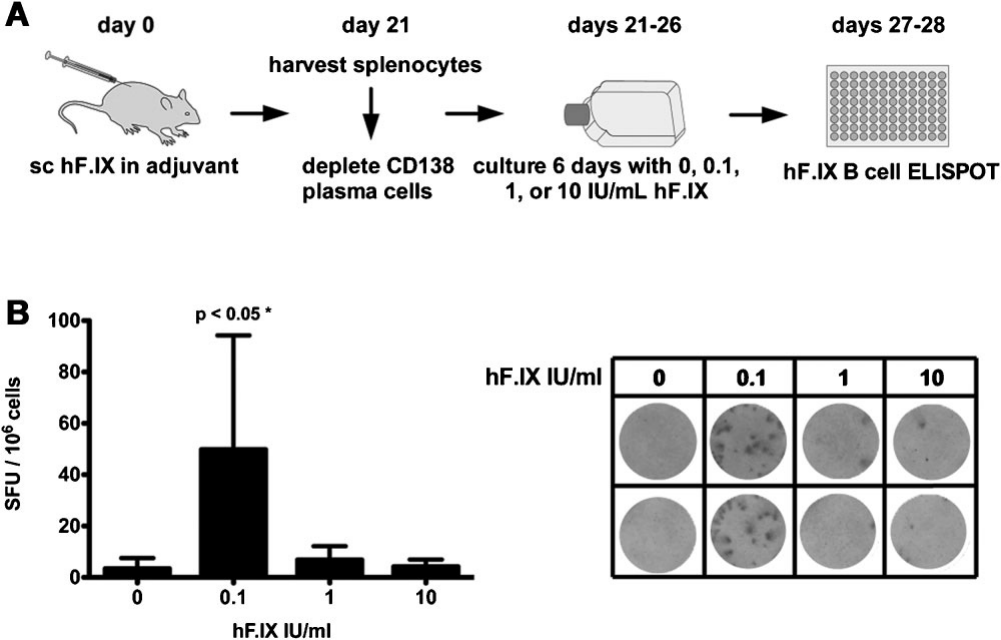
Levels of hFIX antigen impact activation of memory B cells Haemophilia B mice were immunized with hFIX in adjuvant followed by splenocyte isolation 3 weeks later. Plasma cells were depleted using CD138 beads from Miltenyi Biotech, and cells were cultured for 6 days with 0, 0.1, 1 or 10 IU/ml hFIX protein.hFIX-specific B-cell ELISpot was performed on cells following 6 days of culture and results are reported as spot forming units (SFU)/10^6^ cells with a representative experiment shown to the right. Statistical analysis was performed using one-way ANOVA (*p* = 0.0051) with Bonferroni's multiple comparison test (*p* < 0.05 as a cut off for significance and confidence levels). Data are averaged from three independent experiments using pooled splenocytes from four mice immunized with hFIX protein. Data in bar graph are average ± SD.

Previous studies in a murine haemophilia A model showed that high levels of hF.VIII protein suppress activation of memory B cells (Hausl et al, 2005). We hypothesized that hFIX-specific memory B cells may respond in a similar manner. Splenocytes isolated from C3H/HeJ F9^−/−^ mice that had been immunized against hFIX were depleted of plasma cells by magnetic-activated cell sorting using CD138 beads. Cells were cultured for 6 days with 0, 0.1, 1 and 10 IU/ml hFIX protein, and activation of memory B cells was assessed using a B-cell ELISpot (Fig 5A). No spots were detected without hFIX stimulation, demonstrating effective depletion of plasma cells. Low antigen (0.1 IU/ml hFIX) stimulated memory B cells, which was not seen at higher levels (1–10 IU/ml, Fig 5B). Thus, the effectiveness of higher vector doses may in part be explained by suppression of memory B cells at elevated antigen doses.

### Treg-mediated suppression of antibody formation is vector dose dependent

**Figure 6:**
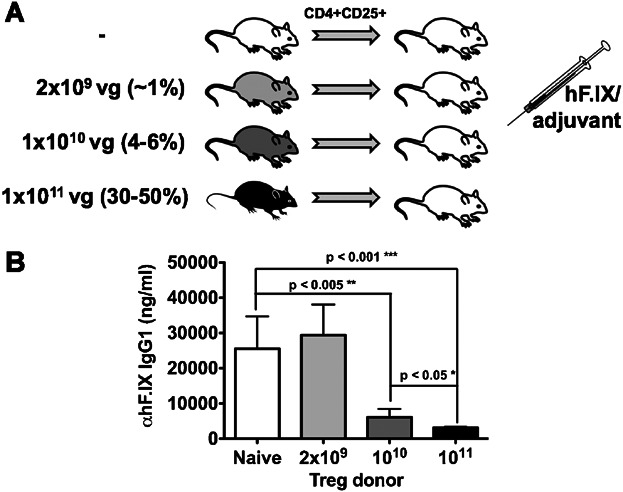
Treg-mediated suppression of anti-hFIX formation is vector dose dependent Vector injected (2 × 10^9^, 1 × 10^10^ or 1 × 10^11^ vg AAV8-ApoE/hAAT *hF9*) C3H/HeJ and naïve mice were Treg donors. Following purification using magnetic activated cell sorting 1 × 10^6^ Treg were injected into naïve recipient C3H/HeJ mice which were challenged the following by with 1 IU hFIX protein in adjuvant.Anti-hFIX IgG1 (ng/ml) titres 3 weeks following subcutaneous challenge of hFIX in adjuvant. Data (average ± SD) is from vector treated Treg (*n* = 4 per vector dose) and naïve Treg controls (*n* = 8). The Student's 2-tailed T test was used to determine statistical significance (naïve *vs*. 10^10^ *p* = 0.0022, naïve *vs*. 10^11^ *p* = 0.0008 and 10^10^ *vs*. 10^11^ *p* = 0.0286).

To test whether Treg induction by gene transfer was vector-dose dependent, C3H/HeJ mice received 1 × 10^11^, 1 × 10^10^ or 2 × 10^9^ vg of AAV8-*hF9* vector, followed by adoptive transfer of 1 × 10^6^ splenic CD4^+^CD25^+^ Treg to naive recipients of the same strain (Fig 6A). Recipient mice were immunized with hFIX in adjuvant on the following day. Compared to naïve control Treg, Treg from mid- and high-dose treated mice significantly suppressed anti-hFIX formation, with Treg from the high-dose group showing the highest degrees of suppression (Fig 6B). In contrast, Treg from low-dose treated mice failed to suppress (Fig 6B).

### Continued suppression by Treg is required for long-term tolerance

**Figure 7:**
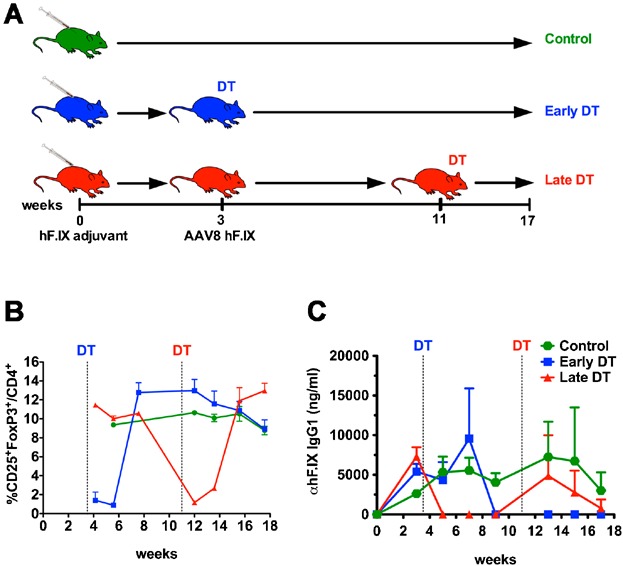
Continued suppression by Treg is required for long-term tolerance Three groups of DTR-FoxP3 mice were challenged with 1 IU hFIX in adjuvant and divided into control, no vector, (green), early DT, DT immediately following vector (blue), and late DT, DT two months following vector (red).Mice were bled at the indicated time points for flow cytometry measurement of %CD25^+^FoxP3^+^/CD4^+^Treg.Anti-hFIX IgG1 titers (ng/ml) as a function of time. Vertical lines indicate respective times of DT administration for early (blue) and late (red) groups. Data points in B-C are average ± SD; *n* = 3–5/experimental group.

To better define the role of Treg in reversal of antibody production against hFIX, we used a diphtheria toxin receptor (DTR) DTR-FoxP3 transgenic mouse model. These mice express the DTR under control of the FoxP3 promoter, allowing for selective killing of Treg following exposure to diphtheria toxin (DT). Because hFIX is less immunogenic in C57BL/6 mice (Lozier et al, 2005), the background strain of DTR-FoxP3 mice, we immunized with hFIX in adjuvant. In a pilot study, WT C57BL/6 mice showed a complete loss of circulating antibodies following high-dose AAV8-*hF9* gene transfer similar to the haemophilic mouse studies (data not shown). Next, DTR-FoxP3 mice were immunized against hFIX and transduced with AAV8-*hF9* vector (1 × 10^11^ vg/mouse) 1 month later. Treg were depleted by DT administration on days 4 and 8 post-gene transfer (‘early DT’ group) or 2 months after gene transfer (‘late DT’ group, again two doses, 4 days apart) (Fig 7A). Flow cytometry on peripheral blood cells confirmed effective depletion of Treg, which fully recovered within 1 month (Supporting Information Fig S2 and Fig 7B).

Without Treg depletion, vector treatment caused a complete loss of hFIX antibodies within 2 weeks (‘late DT’ group), while anti-hFIX persisted in mice that underwent Treg depletion early after vector treatment (Fig 7C). Interestingly, as Treg recovered (Fig 7B), hFIX antibodies declined to undetectable levels (Fig 7C). During this entire time, DTR-FoxP3 control mice (immunized against hFIX, no vector treatment) showed persistent antibody levels (Fig 7C). Therefore, reversal of anti-hFIX by gene transfer was Treg-dependent. Next, we asked whether Treg were still required once antibodies had been eliminated. In the ‘late DT’ group, Treg depletion resulted in re-emergence of antibodies against hFIX, which again returned to undetectable levels following Treg recovery (Fig 7B and C). B-cell ELISpot confirmed absence of splenic ASC of ‘early’ and ‘late’ DT groups by the end of the experiment. Interestingly, one animal in the ‘late DT’ group that had slower kinetics of Treg recovery scored positive for hFIX ACS (Supporting Information Fig S3). Together these results show that there is a constant requirement for Treg to maintain B-cell unresponsiveness.

## DISCUSSION

### A murine haemophilia B model to assess immune responses to recombinant FIX

The C3H/HeJ *F9*^−/−^ model shows a *F9* genotype dependency of inhibitor formation and anaphylaxis that closely mirrors the experience for treatment of human subjects with severe haemophilia B, who are at elevated risk with gene deletions as opposed to mutations that retain endogenous coding information (Verma et al, 2010). These results are also consistent with our prior data on the influence of the underlying *F9* mutation on B- and T-cell responses in gene therapy (Cao et al, 2009). This murine model may serve to determine the relative risks of immunity and anaphylaxis to new recombinant hFIX products including those affecting hFIX half-life in circulation (Metzner et al, 2009; Ostergaard et al, 2011; Peters et al, 2010) and mutants with higher specific activity such as hFIX (R338L) Padua, which has 8–10-fold higher specific activity compared to wild-type hFIX (Simioni et al, 2009). Our results on hFIX dose affects would predict that such a more active variant may avoid anaphylaxis and reduce but perhaps not eliminate inhibitor formation.

### Effective elimination of pathogenic antibodies against FIX—clinical implications

While the incidence of inhibitors in treatment of haemophilia B patient is low compared to haemophilia A, these tend to be high-titre, associated with anaphylactic reactions, and difficult to eradicate. We were able to restore haemostasis in animals with inhibitors and high risk of fatal anaphylactic reactions to FIX. Furthermore, our study demonstrates safe, effective and rapid elimination of these inhibitors without use of immune suppressive drugs. Lack of anaphylaxis after gene transfer suggests that secretion of hFIX by hepatocytes is qualitatively different from a bolus injection of protein and does not result in antibody-antigen complexes triggering the allergic response. Circulating IgG and IgE became undetectable, and antibody production by B cells ceased. While the former is important for elimination of anaphylaxis, the latter is important for avoidance of nephrotic syndrome. If hFIX production and antibody formation occurred simultaneously for a prolonged period of time, continuous presence of hFIX-antibody complexes could cause serious kidney damage (Ewenstein et al, 1997). Should tolerance induction by hepatic gene transfer fail while transduced hepatocytes continued to express hFIX, recently developed transient immune suppression regimens could be implemented as a second line of inhibitor treatment. In non-human primate studies, rituximab combined with cyclophosphamide or cyclosporine has been reproducibly effective for this purpose, and experience with such a protocol is also emerging in human treatment (Mingozzi et al, 2012; Nathwani et al, 2006; Verghese et al, 2013). The fact that we are able to resume hFIX protein injections in tolerized animals without relapse of inhibitors or allergic reactions has additional implications for human treatment, as supplementary protein therapy may be required on occasion even in patients with mild disease.

Although a number of studies have now shown that liver-directed transgene expression from an AAV vector results in transgene tolerance, until now few data had been available on gene transfer with pre-existing immunity against the transgene product (Cao et al, 2007; LoDuca et al, 2009; Mingozzi et al, 2003; Niemeyer et al, 2009). A recently published study showed inhibitor reversal following AAV liver gene transfer of canine F.VIII to haemophilia A dogs with low-titre inhibitors (Finn et al, 2010). Our study shows reversal of antibody formation in haemophilic animals with high-titre inhibitors. Clinically, titres above 5 Bethesda unit (BU) are considered high, because haemostasis cannot be restored by treatment with factor even if doses are adjusted. Instead, bypassing agents have to be used. In our study, the time course of reversal of inhibitors was quite short but also vector dose-dependent. Using hepatic gene transfer in naïve animals, we repeatedly found that expression of approximately 1% of normal circulating hFIX levels represented a threshold, at which the balance started to tip toward tolerance. Here, we reliably reversed inhibitors at the mid-dose, resulting in average expression of 4–6% of normal. At a fivefold lower dose, *i.e*. at the ∼1% expression level, we failed. Therefore, the minimal expression level required for inhibitor reversal is slightly higher but still similar to that required for prevention of inhibitor formation in previously untreated animals. Encouragingly for translation, FIX levels of approximately 6% of normal have now been obtained in several haemophilia B patients (Davidoff et al, 2012). Future pre-clinical studies should determine whether there is a limit in the inhibitor titre for reversal by gene transfer and whether vector doses may have to be adjusted depending on the pre-existing titre.

### B cells, T cells and Treg—a tale of ongoing suppression

Inhibition of memory B-cell responses by high antigen doses, as shown here for hFIX, may represent a general phenomenon for soluble protein antigen, since similar findings have been reported for F.VIII (Hausl et al, 2005), and in part explains the mechanism of current ITI protocols for haemophilia. While high levels of hFIX protein may also account for reduced activation of memory B cells at higher levels of hFIX expression in our protocol, plasma cells, responsible for the majority of ongoing antibody production, are not affected. Therefore additional mechanisms are likely at play. There is strong evidence that the development and maintenance of inhibitors in haemophilia requires T helper cell dependent B-cell activation (Bray et al, 1993; Cao et al, 2006; Fields et al, 2000). Previously, we have provided direct evidence for tolerization of transgene product-specific CD4^+^ T cells upon hepatic gene transfer and for suppression of T help and antibody formation by induction of CD4^+^CD25^+^FoxP3^+^ Treg (Cao et al, 2007; Dobrzynski et al, 2004, 2006; Mingozzi et al, 2003). The current study shows improved Treg induction with increased vector doses. While these results formally do not differentiate between higher numbers and increased suppressive phenotype of induced Treg, we have previously provided evidence that higher expression levels lead to increased frequency of transgene product-specific Treg (Cooper et al, 2009). The fact that we are able to demonstrate Treg induction in adoptive transfer studies but not upon *in vitro* re-stimulation with hFIX (which revealed elimination of T helper responses in tolerized animals) is consistent with our prior data. Compared to other approaches such as co-administration of antigen and rapamycin, AAV gene transfer induced antigen-specific Treg are present at low frequency but are nonetheless critical for immune tolerance (Cao et al, 2007; Dobrzynski et al, 2006; Moghimi et al, 2011; Nayak et al, 2009, 2011).

In summary, high levels of hFIX expression likely direct reversal of inhibitor formation through direct (inhibition of *B*_M_) and indirect mechanisms of B-cell suppression (induction of Treg and elimination of T help). Several studies have shown that Treg can directly interact with B cells and—in a contact dependent manner—either suppress (Iikuni et al, 2009; Lim et al, 2005) or selectively kill (Zhao et al, 2006). Further, Treg have been shown to suppress development of long-lived plasma cells (Jang et al, 2011). Our study also points to a prominent role of Treg for regulating humoural immunity. Experiments in the conditional Treg knockout strain (FoxP3-DTR) indicate the Treg are not only required during the initial phase of inhibitor reversal but also to maintain tolerance subsequently. In fact, anti-hFIX responses relapse quickly if Treg are depleted after tolerance has been established. Therefore, direct inhibition of *B*_M_ by hFIX or Treg-mediated deletion of B cells may contribute to but cannot entirely explain elimination of antibody production. Rather, the data suggest that hFIX-reactive B cells persist in a suppressed state, so that continuous suppression by Treg is required to prevent relapse of antibodies. The robustness of this regulatory response is remarkable, given that B-cell tolerance is rapidly regained as Treg recover. The data from this model do not rule out a contribution by Treg that are not hFIX-specific, albeit induced antigen-specific Treg are likely critical for suppression (Annoni et al, 2013; Cao et al, 2007; Matrai et al, 2011).

How antigen derived from hepatocytes is presented to MHC class II-restricted CD4^+^ T cells in a tolerogenic fashion, leading to deletion of effector T cells and induction of Treg, is a question that requires further study. In a first set of experiments, we find that antigen presentation leading to proliferation of transgene product-specific CD4^+^ T cells is dependent on professional APCs, including macrophages and dendritic cells (see Supporting Information Fig S4). These data do not entirely prove but at least support a model of professional APCs playing an intermediary role in cross presentation of hepatocyte-derived antigen to CD4^+^ T cells.

### Alternative vector platforms for gene transfer-based ITI

We have established a first animal model of anaphylaxis in FIX replacement therapy that resembles the outcomes of replacement therapy in the haemophilia B patient population. Hepatic AAV-*hF9* gene transfer safely and effectively restored haemostasis in these animals. Suppression of B-cell responses resulted in rapid reversal of inhibitor formation and of IgE formation, thereby desensitizing from anaphylaxis, so that supplementary protein therapy could safely be performed. Absence not only of circulating antibodies but also of cellular antibody production should alleviate concerns that the approach could lead to nephrotic syndrome, albeit protocols for immune suppression regiments should be in place as a fallback safety measure. Regimens that deplete Treg should be avoided as these play a critical role for maintaining suppression and preventing relapse of antibodies. Such considerations may also be important if a patient was going to receive an organ transplant at some point later in life. Hepatic gene transfer as a means of reversing pathogenic antibody formation is also applicable to lysosomal storage disorders as shown for Pompe disease (Sun et al, 2010). Reversal of high-tier F.VIII inhibitors in congenital haemophilia A likely requires further optimization of hepatic gene expression (McIntosh et al, 2013; Sack et al, 2012; Ward et al, 2011). Finally, alternative vector systems such as miRNA-regulated lentiviral vectors (Brown et al, 2007; Matrai et al, 2011), which have been optimized for Treg induction upon hepatic gene transfer, are being developed to increase our tool kit for gene transfer-based protocols that may in the future replace current ITI.

**The paper explained**

**PROBLEM:**

Protein replacement therapies for genetic diseases are often severely complicated by antibody formation, which may also be associated with severe allergic reactions. One example is antibody formation against FIX in treatment of haemophilia B. These ‘inhibitors’ often cannot be eradicated using current immune tolerance induction (ITI) protocols because of anaphylaxis or nephrotic syndrome upon prolonged exposure to FIX. Alternative tolerance protocols are urgently needed to alleviate the high risk for morbidity and mortality in these patients.

**RESULTS:**

Hepatic adeno-associated viral *F9* gene transfer efficiently and rapidly reversed high-titre inhibitors and provided long-term correction of haemostasis in a murine haemophilia B. Tolerized animals were protected from subsequent anaphylactic reactions against FIX. Reversal was vector dose dependent. High FIX antigen doses suppressed activation of memory B cells, induced regulatory T cells (Treg), and eliminated existing anti-FIX formation by B cells. Persistent suppression by Treg was required to prevent relapse of antibody formation.

**IMPACT:**

Together these data demonstrate that hepatic gene transfer-based ITI provides a safe and effective alternative to eradicate inhibitors even in the face of anaphylaxis, supporting the suitability of the protocol for translational studies. This approach should be applicable to reverse pathogenic antibodies in other enzyme replacement therapies and may also be able to treat autoimmunity caused by auto-antibodies.

Indeed, using a lentiviral vector Annoni et al in a companion manuscript demonstrate independently that liver gene transfer reverses pre-existing FIX inhibitors in haemophilia B mice while avoiding the severe allergic reactions that plague current ITI protocols (that are based on IV injections of factor product) (Annoni et al, 2013). Both our studies are in agreement in that shut-down of antibody production by B cells via Treg induction and suppression of memory B cells are major mechanisms, which supports further translational studies. While we were able to effectively reverse FIX inhibitors with ∼6% normal levels FIX protein, Annoni et al observed a requirement for higher 50–100% normal levels FIX protein for effective reversal. It is important to note that there are several differences in our models such as, the background strain of the haemophilia B mice (C3H/HeJ *vs*. C57BL/6) and immunization protocols (IV FIX protein *vs*. FIX protein in adjuvant), antibody titres (30 *vs*. 200 μg/ml) and relative vector immunity (AAV *vs*. lentivirus) that may explain the different requirements for FIX expression to effectively reverse inhibitors. Nonetheless both these studies demonstrate that different vector systems can be adapted to take advantage of tolerogenic expression by hepatocytes to treat FIX inhibitors.

## MATERIALS AND METHODS

### Viral vectors

AAV-ApoE/hAAT-*hF9* carries the hepatocyte-specific expression cassette for hFIX (Manno et al, 2006). This cassette includes an apolipoprotein E (ApoE) enhancer/hepatocyte control region, a human a1-antitrypsin promoter, hFIX cDNA, a 1.4-kb portion of intron I of the *F9* gene, and the bovine growth hormone poly(A) signal. AAV serotype 8 vector was produced as previously described (Cooper et al, 2009; Liu et al, 2003).

### Animal studies

C3H/HeJ and C57BL/6 mice were purchased from Jackson Laboratories (Bar Harbor, ME). Haemophilia B mice with targeted deletion of murine *F9* (‘Null mutation’) had been bred on C3H/HeJ background for >10 generations (Mingozzi et al, 2003). Mice transgenic for *hF9* variants (human *F9* complementary DNA including a 0.3-kb portion of intron I expressed from liver-specific transthyretin promoter) were as published (Sabatino et al, 2004). These animals express hFIX with late stop codon at amino acid residue 338 (‘LS’, crim−); crim− G381E missense mutation (‘CH’, identical mutation as in haemophilia B dogs at University of North Carolina Chapel Hill); or crim+ R180W missense mutation. These lines were originally numbered as LS-37, cCH-6 and MS-12, and contain 6, 10 and 1 copy of the human *F9* gene, respectively (Sabatino et al, 2004). The lines were repeatedly backcrossed onto C3H/HeJ background (>10 generation), and finally crossed with Null mice in order to eliminate endogenous murine *F9* expression (Cao et al, 2009). C57BL/6 DTR-FoxP3 mice were kindly provided by Dr. Alexander Rudensky (Kim et al, 2007). Animals were housed under special pathogen-free conditions at the University of Florida and treated under Institutional Animal Care and Use Committee-approved protocols. All animals were male and 6–8 weeks old at the onset of the experiments.

Immunization protocols for haemophilia B mice were performed as described unless otherwise stated (Verma et al, 2010). Briefly, mice were given six weekly injections of recombinant hFIX protein (Benefix, 1 IU/dose) starting with an intraperitoneal injection and followed by five weekly intraveneous injections into the tail vein. Plasma samples were collected by tail bleed into citrate buffer as described (Mingozzi et al, 2003). For adoptive transfer and Treg depletion studies, mice were bled from the retro-orbital plexus using heparinized microcapillary tubes. Where indicated mice received hFIX protein along with 150 µg antihistamine (triprolidine; Sigma) and 50 µg platelet-activating factor (PAF) antagonist CV-3988 to prevent anaphylactic responses. In some studies mice were immunized with 1 IU hFIX protein in adjuvant (Sigma Adjuvant System S6322, Sigma, St. Louis, MO).

### Antigen and antibody measurements

Plasma levels of hFIX antigen were measured by ELISA and inhibitory antibody titres were determined by Bethesda assay as published (Cao et al, 2009; Mingozzi et al, 2003). One Bethesda unit is the reciprocal of the dilution of plasma that neutralizes 50% of the FIX in a normal plasma sample which it is mixed *in vitro*. Immunocapture assays to determine titres of hFIX-specific IgGE and IgG1 were as described (Cao et al, 2009; Mingozzi et al, 2003) using purified mouse IgE and IgG1 as standards. To measure IgE titres, IgG was removed from plasma samples using protein G sepharose (GE Healthcare).

### B- and T-cell assays

B-cell ELISpots were performed on total splenocytes and bone marrow cells as previously described (Hausl et al, 2002, 2004; Wang et al, 2005). In some studies, plasma cells were depleted using anti mouse CD138 beads (Miltenyi, Vancouver, Canada) according to manufacturers instructions. For cytokine response studies, isolated splenocytes were cultured in RPMI 1640 media (containing 50 µM β-mercaptoethanol, 100 mM insulin/transferrin/selenium, glutamine and antibiotics) with or without hFIX 10 μg/ml for 48 h (37°C, 5% CO_2_). Transcript levels of cytokines were measured by quantitative RT-PCR using SA Biosciences arrays (Frederick, MD, USA; RNA was extracted from 1.5 × 10^5^ cells per spleen prior to cDNA synthesis) and a MyQ thermocycler (Biorad, Hercules, CA, USA), and normalized based on GAPDH expression.

### Adoptive T-cell transfer studies

CD4 + CD25+ splenocytes from vector treated or naïve C3H/HeJ mice were purified using the Treg magnetic activated cell sorting kit from Miltenyi, pooled, and adoptively transferred to naïve mice of the same strain by tail vein injection (1 × 10^6^ cells per mouse). Recipient mice were immunized by subcutaneous injection of 1 IU hFIX (in Sigma Adjuvant System) 24 h later.

### Treg depletion studies

DTR-FoxP3 mice were immunized by subcutaneous injection of 1 IU hFIX (in Sigma Adjuvant System). Three weeks post-immunization mice were injected with 1 × 10^11^ vg of an AAV8 ApoE/hAAT *hF9* vector in the tail vein and split into two groups of early and late DT treatment. Mice received two IP injections of DT 50 μg/kg with a 4-day interval. Depletion and recovery of Treg was monitored by flow cytometry of peripheral blood using anti mouse CD3-PerCP-Cy5.5 (BD Biosciences), CD4-e450 (eBioscience), CD25-PE (eBioscience) and FoxP3-Alexa647 (eBioscience).

### Statistical Analysis

All statistical analysis was carried out using Prism software using Student's 2-tailed *T*-test or one-way ANOVA with Bonferroni's multiple comparison test. A *p* < 0.05 was considered statistically significant.

## Supplementary material

As a service to our authors and readers, this journal provides supporting information supplied by the authors. Such materials are peer reviewed and may be re-organized for online delivery, but are not copy-edited or typeset. Technical support issues arising from supporting information (other than missing files) should be addressed to the authors.

**Figure S1. AAV8-ApoEe/hAAT-*hF9* bio-distribution and liver specific expression of hFIX protein.** Vector was given by tail vein injection at 1 × 10^11^ vg/mouse to haemophilia B mice (C3H/HeJ *F9*^−/−^). (A) AAV8-ApoEe/hAAT-*hF9* bio-distribution performed on genomic DNA isolated from liver, kidney, lung and spleen 3 weeks following IV administration in C3H/HeJ *F9*^−/−^ mice (*n* = 3). (B) *hF9* mRNA expression determined by RT-qPCR performed on total RNA isolated from liver, kidney, lung and spleen 3 weeks following IV administration in C3H/HeJ *F9*^−/−^ mice (*n* = 3). GAPDH copy numbers in black (left axis) and *hF9* copy numbers in grey (right axis). A no reverse transcriptase control was included for liver RNA samples (*n* = 3). (C) Representative liver section stained for hFIX (red) and F4/80 Kupffer cells and macrophages (green). (D) Representative spleen section stained for hFIX (red) and F4/80 macrophages (green).

**Figure S2. Recovery of Treg following two doses of DT.** (A) Gating scheme of mouse PBMC using CD3-PerCP-CY5.5 and CD4-e450 gates to select CD4 + T cells. (B) Representative dot plots used to calculate %CD25 + FoxP3 + Treg for control untreated DTR-FoxP3 mice (left column) and DT treated DTR-FoxP3 mice (right column) with day 0 as the first DT injection.

**Figure S3. Residual hF.IX ASC detected in a ‘late DT**’ **mouse with delayed Treg reconstitution.** (A) Flow cytometry staining of peripheral blood 14 days following late DT treatment. (B) hF.IX B-cell ELISpot on splenocytes from late DT treated mice isolated 29 days post-DT treatment.

**Figure S4. Requirement for professional antigen presenting cells (APCs) for transgene product-specific CD4**^**+**^ **T-cell proliferation.** AAV8-EF1α-ova vector was administered by tail vein injection to BALB/c mice at 1 × 10^11^ vg/mouse. (A) Proliferation of transferred T cells in spleens and livers of BALB/c mice. Left panel: control animal. Right panel: animal, in which Kupffer cells and M1 macrophages were inactivated using gadolinium chloride (GdCl_3_). (B) Proliferation of transferred T cells in spleens and livers of conditional CD11c deficient mice (CD11-DTR BALB/c mice). Left panel: control animal. Right panel: animal, in which CD11c^+^ DCs cells were depleted following administration of diphtheria toxin (DT). Shown are representative examples for *n* = 3 per experimental group. Percent cells that have or have not proliferated are indicated. No proliferation was observed in BALB/c mice that had not received AAV-ova vector (data not shown).

## References

[b1] Annoni A, Cantore A, Della Valle P, Goudy K, Akbarpour M, Russo F, Bartolaccini S, D'Angelo A, Roncarolo MG, Naldini L (2013). Liver gene therapy by lentiviral vectors reverses anti-Factor IX pre-existing immunity in hemophilic mice. EMBO Mol Med.

[b2] Bray GL, Kroner BL, Arkin S, Aledort LW, Hilgartner MW, Eyster ME, Ragni MV, Goedert JJ (1993). Loss of high-responder inhibitors in patients with severe hemophilia A and human immunodeficiency virus type 1 infection: a report from the Multi-Center Hemophilia Cohort Study. Am J Hematol.

[b3] Brown BD, Cantore A, Annoni A, Sergi LS, Lombardo A, Della Valle P, D'Angelo A, Naldini L (2007). A microRNA-regulated lentiviral vector mediates stable correction of hemophilia B mice. Blood.

[b4] Cao O, Armstrong E, Schlachterman A, Wang L, Okita DK, Conti-Fine B, High KA, Herzog RW (2006). Immune deviation by mucosal antigen administration suppresses gene-transfer-induced inhibitor formation to factor IX. Blood.

[b5] Cao O, Dobrzynski E, Wang L, Nayak S, Mingle B, Terhorst C, Herzog RW (2007). Induction and role of regulatory CD4+CD25+ T cells in tolerance to the transgene product following hepatic in vivo gene transfer. Blood.

[b6] Cao O, Hoffman BE, Moghimi B, Nayak S, Cooper M, Zhou S, Ertl HC, High KA, Herzog RW (2009). Impact of the underlying mutation and the route of vector administration on immune responses to factor IX in gene therapy for hemophilia B. Mol Ther.

[b7] Chitlur M, Warrier I, Rajpurkar M, Lusher JM (2009). Inhibitors in factor IX deficiency a report of the ISTH-SSC international FIX inhibitor registry (1997–2006). Haemophilia.

[b8] Cooper M, Nayak S, Hoffman BE, Terhorst C, Cao O, Herzog RW (2009). Improved induction of immune tolerance to factor IX by hepatic AAV-8 gene transfer. Hum Gene Ther.

[b9] Davidoff A, Tuddenham EG, Rangarajan S, Rosales C, McIntosh J, Chowdary P, Riddell A, Glader B, Rustagi P, Ng C (2012). Stable factor IX activity following AAV-mediated gene transfer in patients with severe hemophilia B. ASH Annu Meet Abstr.

[b10] DiMichele D (2007). Inhibitor development in haemophilia B: an orphan disease in need of attention. Br J Haematol.

[b11] DiMichele DM (2012). Immune tolerance in haemophilia: the long journey to the fork in the road. Br J Haematol.

[b12] Dobrzynski E, Fitzgerald JC, Cao O, Mingozzi F, Wang L, Herzog RW (2006). Prevention of cytotoxic T lymphocyte responses to factor IX-expressing hepatocytes by gene transfer-induced regulatory T cells. Proc Natl Acad Sci USA.

[b13] Dobrzynski E, Mingozzi F, Liu YL, Bendo E, Cao O, Wang L, Herzog RW (2004). Induction of antigen-specific CD4+ T-cell anergy and deletion by in vivo viral gene transfer. Blood.

[b14] Ewenstein BM, Takemoto C, Warrier I, Lusher J, Saidi P, Eisele J, Ettinger LJ, DiMichele D (1997). Nephrotic syndrome as a complication of immune tolerance in hemophilia B. Blood.

[b15] Fields PA, Kowalczyk DW, Arruda VR, Armstrong E, McCleland ML, Hagstrom JN, Pasi KJ, Ertl HC, Herzog RW, High KA (2000). Role of vector in activation of T cell subsets in immune responses against the secreted transgene product factor IX. Mol Ther.

[b16] Finn JD, Ozelo MC, Sabatino DE, Franck HW, Merricks EP, Crudele JM, Zhou S, Kazazian HH, Lillicrap D, Nichols TC (2010). Eradication of neutralizing antibodies to factor VIII in canine hemophilia A after liver gene therapy. Blood.

[b17] Hausl C, Ahmad RU, Sasgary M, Doering CB, Lollar P, Richter G, Schwarz HP, Turecek PL, Reipert BM (2005). High-dose factor VIII inhibits factor VIII-specific memory B cells in hemophilia A with factor VIII inhibitors. Blood.

[b18] Hausl C, Ahmad RU, Schwarz HP, Muchitsch EM, Turecek PL, Dorner F, Reipert BM (2004). Preventing restimulation of memory B cells in hemophilia A: a potential new strategy for the treatment of antibody-dependent immune disorders. Blood.

[b19] Hausl C, Maier E, Schwarz HP, Ahmad RU, Turecek PL, Dorner F, Reipert BM (2002). Long-term persistence of anti-factor VIII antibody-secreting cells in hemophilic mice after treatment with human factor VIII. Thromb Haemost.

[b20] Hoffman BE, Dobrzynski E, Wang L, Hirao L, Mingozzi F, Cao O, Herzog RW (2007). Muscle as a target for supplementary factor IX gene transfer. Hum Gene Ther.

[b21] Hoffman BE, Martino AT, Sack BK, Cao O, Liao G, Terhorst C, Herzog RW (2011). Nonredundant roles of IL-10 and TGF-β in suppression of immune responses to hepatic AAV-factor IX gene transfer. Mol Ther.

[b22] Iikuni N, Lourenco EV, Hahn BH, La Cava A (2009). Cutting edge: regulatory T cells directly suppress B cells in systemic lupus erythematosus. J Immunol.

[b23] Jadhav M, Warrier I (2000). Anaphylaxis in patients with hemophilia. Semin Thromb Hemost.

[b24] Jang E, Cho WS, Cho ML, Park HJ, Oh HJ, Kang SM, Paik DJ, Youn J (2011). Foxp3+ regulatory T cells control humoral autoimmunity by suppressing the development of long-lived plasma cells. J Immunol.

[b25] Kim JM, Rasmussen JP, Rudensky AY (2007). Regulatory T cells prevent catastrophic autoimmunity throughout the lifespan of mice. Nat Immunol.

[b26] Koeberl DD, Kishnani PS (2009). Immunomodulatory gene therapy in lysosomal storage disorders. Curr Gene Ther.

[b27] Lim HW, Hillsamer P, Banham AH, Kim CH (2005). Cutting edge: direct suppression of B cells by CD4+ CD25+ regulatory T cells. J Immunol.

[b28] Liu YL, Wagner K, Robinson N, Sabatino D, Margaritis P, Xiao W, Herzog RW (2003). Optimized production of high-titer recombinant adeno-associated virus in roller bottles. BioTechniques.

[b29] LoDuca PA, Hoffman BE, Herzog RW (2009). Hepatic gene transfer as a means of tolerance induction to transgene products. Curr Gene Ther.

[b30] Lozier JN, Tayebi N, Zhang P (2005). Mapping of genes that control the antibody response to human factor IX in mice. Blood.

[b31] Lu Y, Song S (2009). Distinct immune responses to transgene products from rAAV1 and rAAV8 vectors. Proc Natl Acad Sci USA.

[b32] Manno CS, Pierce GF, Arruda VR, Glader B, Ragni M, Rasko JJ, Ozelo MC, Hoots K, Blatt P, Konkle B (2006). Successful transduction of liver in hemophilia by AAV-Factor IX and limitations imposed by the host immune response. Nat Med.

[b33] Markusic DM, Herzog RW, Aslanidi GV, Hoffman BE, Li B, Li M, Jayandharan GR, Ling C, Zolotukhin I, Ma W (2010). High-efficiency transduction and correction of murine hemophilia B using AAV2 vectors devoid of multiple surface-exposed tyrosines. Mol Ther.

[b34] Martino AT, Basner-Tschakarjan E, Markusic DM, Finn JD, Hinderer C, Zhou S, Ostrov DA, Srivastava A, Ertl HC, Terhorst C (2013). Engineered AAV vector minimizes in vivo targeting of transduced hepatocytes by capsid-specific CD8+ T cells. Blood.

[b35] Matrai J, Cantore A, Bartholomae CC, Annoni A, Wang W, Acosta-Sanchez A, Samara-Kuko E, De Waele L, Ma L, Genovese P (2011). Hepatocyte-targeted expression by integrase-defective lentiviral vectors induces antigen-specific tolerance in mice with low genotoxic risk. Hepatology.

[b36] McIntosh J, Lenting PJ, Rosales C, Lee D, Rabbanian S, Raj D, Patel N, Tuddenham EG, Christophe OD, McVey JH (2013). Therapeutic levels of FVIII following a single peripheral vein administration of rAAV vector encoding a novel human factor VIII variant. Blood.

[b37] Metzner HJ, Weimer T, Kronthaler U, Lang W, Schulte S (2009). Genetic fusion to albumin improves the pharmacokinetic properties of factor IX. Thromb Haemost.

[b38] Mingozzi F, Chen Y, Murphy SL, Edmonson SC, Tai A, Price SD, Metzger ME, Zhou S, Wright JF, Donahue RE (2012). Pharmacological modulation of humoral immunity in a nonhuman primate model of AAV gene transfer for hemophilia B. Mol Ther.

[b39] Mingozzi F, Liu YL, Dobrzynski E, Kaufhold A, Liu JH, Wang Y, Arruda VR, High KA, Herzog RW (2003). Induction of immune tolerance to coagulation factor IX antigen by in vivo hepatic gene transfer. J Clin Invest.

[b40] Moghimi B, Sack BK, Nayak S, Markusic DM, Mah CS, Herzog RW (2011). Induction of tolerance to factor VIII by transient co-administration with rapamycin. J Thromb Haemost.

[b41] Nathwani AC, Gray JT, Ng CY, Zhou J, Spence Y, Waddington SN, Tuddenham EG, Kemball-Cook G, McIntosh J, Boon-Spijker M (2006). Self-complementary adeno-associated virus vectors containing a novel liver-specific human factor IX expression cassette enable highly efficient transduction of murine and nonhuman primate liver. Blood.

[b42] Nathwani AC, Tuddenham EG, Rangarajan S, Rosales C, McIntosh J, Linch DC, Chowdary P, Riddell A, Pie AJ, Harrington C (2011). Adenovirus-associated virus vector-mediated gene transfer in hemophilia B. N Engl J Med.

[b43] Nayak S, Cao O, Hoffman BE, Cooper M, Zhou S, Atkinson MA, Herzog RW (2009). Prophylactic immune tolerance induced by changing the ratio of antigen-specific effector to regulatory T cells. J Thromb Haemost.

[b44] Nayak S, Sarkar D, Perrin GQ, Moghimi B, Hoffman BE, Zhou S, Byrne BJ, Herzog RW (2011). Prevention and reversal of antibody responses against factor IX in gene therapy for hemophilia B. Front Microbiol.

[b45] Niemeyer GP, Herzog RW, Mount J, Arruda VR, Tillson DM, Hathcock J, van Ginkel FW, High KA, Lothrop CD (2009). Long-term correction of inhibitor-prone hemophilia B dogs treated with liver-directed AAV2-mediated factor IX gene therapy. Blood.

[b46] Ostergaard H, Bjelke JR, Hansen L, Petersen LC, Pedersen AA, Elm T, Moller F, Hermit MB, Holm PK, Krogh TN (2011). Prolonged half-life and preserved enzymatic properties of factor IX selectively PEGylated on native N-glycans in the activation peptide. Blood.

[b47] Passini MA, Bu J, Fidler JA, Ziegler RJ, Foley JW, Dodge JC, Yang WW, Clarke J, Taksir TV, Griffiths DA (2007). Combination brain and systemic injections of AAV provide maximal functional and survival benefits in the Niemann-Pick mouse. Proc Natl Acad Sci USA.

[b48] Peters RT, Low SC, Kamphaus GD, Dumont JA, Amari JV, Lu Q, Zarbis-Papastoitsis G, Reidy TJ, Merricks EP, Nichols TC (2010). Prolonged activity of factor IX as a monomeric Fc fusion protein. Blood.

[b49] Recht M, Pollmann H, Tagliaferri A, Musso R, Janco R, Richey Neuman W (2011). A retrospective study to describe the incidence of moderate to severe allergic reactions to factor IX in subjects with haemophilia B. Haemophilia.

[b50] Sabatino DE, Armstrong E, Edmonson S, Liu YL, Pleimes M, Schuettrumpf J, Fitzgerald J, Herzog RW, Arruda VR, High KA (2004). Novel hemophilia B mouse models exhibiting a range of mutations in the Factor IX gene. Blood.

[b51] Sack BK, Merchant S, Markusic DM, Nathwani AC, Davidoff AM, Byrne BJ, Herzog RW (2012). Transient B cell depletion or improved transgene expression by codon optimization promote tolerance to factor VIII in gene therapy. PLoS ONE.

[b52] Simioni P, Tormene D, Tognin G, Gavasso S, Bulato C, Iacobelli NP, Finn JD, Spiezia L, Radu C, Arruda VR (2009). X-linked thrombophilia with a mutant factor IX (factor IX Padua). N Engl J Med.

[b53] Sun B, Kulis MD, Young SP, Hobeika AC, Li S, Bird A, Zhang H, Li Y, Clay TM, Burks W (2010). Immunomodulatory gene therapy prevents antibody formation and lethal hypersensitivity reactions in murine pompe disease. Mol Ther.

[b54] Thorland EC, Drost JB, Lusher JM, Warrier I, Shapiro A, Koerper MA, Dimichele D, Westman J, Key NS, Sommer SS (1999). Anaphylactic response to factor IX replacement therapy in haemophilia B patients: complete gene deletions confer the highest risk. Haemophilia.

[b55] Vandendriessche T, Thorrez L, Acosta-Sanchez A, Petrus I, Wang L, Ma L, DE Waele L, Iwasaki Y, Gillijns V, Wilson JM (2007). Efficacy and safety of adeno-associated viral vectors based on serotype 8 and 9 vs. lentiviral vectors for hemophilia B gene therapy. J Thromb Haemost.

[b56] Verghese P, Darrow S, Kurth MH, Reed RC, Kim Y, Kearney S (2013). Successful management of factor IX inhibitor-associated nephrotic syndrome in a hemophilia B patient. Pediatr Nephrol.

[b57] Verma D, Moghimi B, LoDuca PA, Singh HD, Hoffman BE, Herzog RW, Daniell H (2010). Oral delivery of bioencapsulated coagulation factor IX prevents inhibitor formation and fatal anaphylaxis in hemophilia B mice. Proc Natl Acad Sci USA.

[b58] Wang L, Cao O, Swalm B, Dobrzynski E, Mingozzi F, Herzog RW (2005). Major role of local immune responses in antibody formation to factor IX in AAV gene transfer. Gene Therapy.

[b59] Ward NJ, Buckley SM, Waddington SN, Vandendriessche T, Chuah MK, Nathwani AC, McIntosh J, Tuddenham EG, Kinnon C, Thrasher AJ (2011). Codon optimization of human factor VIII cDNAs leads to high-level expression. Blood.

[b60] Warrier I, Ewenstein BM, Koerper MA, Shapiro A, Key N, DiMichele D, Miller RT, Pasi J, Rivard GE, Sommer SS (1997). Factor IX inhibitors and anaphylaxis in hemophilia B. J Pediatr Hematol Oncol.

[b61] Zhang P, Sun B, Osada T, Rodriguiz R, Yang XY, Luo X, Kemper AR, Clay TM, Koeberl DD (2012). Immunodominant liver-specific expression suppresses transgene-directed immune responses in murine pompe disease. Hum Gene Ther.

[b62] Zhao DM, Thornton AM, DiPaolo RJ, Shevach EM (2006). Activated CD4+CD25+ T cells selectively kill B lymphocytes. Blood.

[b63] Zhong L, Li B, Mah CS, Govindasamy L, Agbandje-McKenna M, Cooper M, Herzog RW, Zolotukhin I, Warrington KH, Weigel-Van Aken KA (2008). Next generation of adeno-associated virus 2 vectors: point mutations in tyrosines lead to high-efficiency transduction at lower doses. Proc Natl Acad Sci USA.

