# Supplementary material for: Effective gene therapy for haemophilic mice with pathogenic factor IX antibodies
Source: EMBO Mol Med. 2013 Sep 16;5(11):1698–709. doi: 10.1002/emmm.201302859 (PMC3840486; doi:10.1002/emmm.201302859)
Supplement: Supplementary file 1 [file emmm0005-1698-SD1.pdf]

EMBO Molecular Medicine

Effective Gene Therapy for Hemophilic Mice with Pathogenic Factor IX Antibodies

David M. Markusic, Brad E. Hoffman, George Q. Perrin, Sushrusha Nayak, Xiaomei Wang, Paul A. LoDuca, Katherine A. High, Roland W. Herzog

Corresponding author: Roland Herzog, University of Florida

Review timeline:

Submission date:	08 April 2013
Editorial Decision:	13 May 2013
Revision received:	18 July 2013
Editorial Decision:	08 August 2013
Revision received:	15 August 2013

Transaction Report:

(Note: With the exception of the correction of typographical or spelling errors that could be a source of ambiguity, letters and reports are not edited. The original formatting of letters and referee reports may not be reflected in this compilation.)

Editor: Roberto Buccione

1st Editorial Decision 13 May 2013

Thank you for the submission of your manuscript to EMBO Molecular Medicine. We have now heard back from the three Reviewers whom we asked to evaluate your manuscript.
You will see that all three Reviewers are generally supportive of you work although Reviewer 1, in particular, expresses some concerns that prevent us from considering publication at this time.

Reviewer 1 would like to understand to what extent transduction of other non-hepatic cells and cross-presentation contribute to tolerisation. S/he also feels that the length of follow-up is limited and thus durability over extended periods should be demonstrated. As Reviewer 1 points out, the manuscript is essentially observational and thus these points need to be fully addressed.

Reviewer 2 is generally satisfied with the manuscript. I should mention that s/he notes in his/her evaluation of back-to-back submission "Liver gene therapy by lentiviral vectors reverses anti-Factor IX pre-existing immunity in hemophilic mice" by Annoni et al. (EMM-2013-02857), that the fact that elimination of neutralising antibodies with AAV-mediated transfer occurred at only 6% of normal FIX levels as reported in this manuscript, is in apparent discrepancy with the other where FIX levels of about 100% of normal appeared to be associated with response. Reviewer 2 feels, and I agree, that this should be addressed and discussed appropriately.

Reviewer 3 lists a number of issues that require your intervention including proper citation of previous work and elucidation of some unclear passages.

Considered all the above, while publication of the paper cannot be considered at this stage, we would be pleased to consider a revised submission, with the understanding that the Reviewers' concerns must be fully addressed, with additional experimental data where appropriate and that acceptance of the manuscript will entail a second round of review.

Please note that it is EMBO Molecular Medicine policy to allow a single round of revision only and that, therefore, acceptance or rejection of the manuscript will depend on the completeness of your responses included in the next, final version of the manuscript.

As you know, EMBO Molecular Medicine has a "scooping protection" policy, whereby similar findings that are published by others during review or revision are not a criterion for rejection. However, I do ask you to get in touch with us after three months if you have not completed your revision, to update us on the status. Please also contact us as soon as possible if similar work is published elsewhere.

I look forward to seeing a revised form of your manuscript as soon as possible.

***** Reviewer's comments *****

Referee #1 (General Remarks):

The authors address the problem of inhibitor-positive hemophilia in a gene therapy mouse model. The authors conclude that AAV-mediated gene therapy has a tolerisation effect through induction of specific Treg cells. These experiments are well-performed, and convincingly demonstrate induction of specific Treg and depletion of memory B cells in vivo. These data are largely observational however, and the immunological mechanism of Treg induction and memory B cell depletion is not elucidated.

1. To what extent does leaky transduction of other non-hepatic cell types (and cross presentation) contribute to the tolerisation process.
2. The length of follow up of experiments is limited. It would be important to show durability over extended periods.

Referee #2 (General Remarks):

This is an interesting study showing that AAV8 vector mediated hFIX expression can reverse pre-existing anti-hFIX antibody responses in mice and thereby eliminate anaphalaxis and neutralizing antibody responses. The mechanism is complex and is shown to be due to induction of Tregs that suppress B cell memory responses and antibody production. It is notable that AAV-mediated suppression of FIX antibodies was possible at FIX levels of about 6% of normal and therefore potentially achievable in human gene therapy trials. Therefore, this work is medically important because it suggests that recently described AAV gene therapy directed at the liver can now be extended to patients with pre-existing neutralizing antibodies. The experiments are well designed and properly controlled and interpreted. The manuscript is clearly written and organized.

Referee #3 (General Remarks):

Markusic et al convincingly show that they can tolerize previously immunized mice with established anti-factor IX antibodies with liver-directed gene transfer using AAV vectors in hemophilia B mice. This is an important problem in humans for various reasons, particularly that factor IX inhibitors prevent proper therapy for bleeding and/or prophylaxis against bleeding, and inhibitors to factor IX can be associated with anaphylaxis and/or nephrotic syndrome in the event of repeat exposure to factor IX. In fact, the anaphylaxis in humans was first noted in patients undergoing standard desensitization with human factor IX proteins. Thus, a safe, effective approach to treating these inhibitors would be welcome.

Issues/Comments/Concerns:

1. Citation for 1 in 20,000 male births statistic in Introduction? More commonly see 1:30,000 elsewhere.
2. May wish to state in Introduction that inhibitors occur predominantly in patients with severe hemophilia B.
3. May wish to mention in Introduction nephrotic syndrome with factor IX ITI (Ewenstein et al, Blood. 1997;89:1115-
1116. Also in Discussion on page 12.
4. Would be curious to know if this C3H/HeJ background strain is one that is immune to toll-receptor mediated septic shock with exposure to endotoxins? See discussion of C3H strain on page 6.
5. With regard to mild IgG response to factor IX in Balb/c mice (page6), this has been shown previously by Lozier et al 2005;105:1029-1035 with strong adjuvantts (adenovirus). Likewise for mention of low reactivity of C57BL/6 mice on page 10.
6. Materials and Methods section, page 19. Definition of Bethesda unit is awkward/incomplete. One BIAU is the reciprocal of the dilution of plasma that neutralizes 50% of the factor IX in a normal plasma sample with which it is mixed in vitro.
7. Figure 1 is unreadable as presented, if my printout is any guide. It needs to be at least twice as large to be readable, and cannot fit into a single column as attempted here. Other Figures are satisfactory; in fact, Figure 5 could be made slightly smaller and not suffer for the effort.

Minor issues:

1. Factor IX is abbreviated "FIX" in most journals, rather than F.IX.
2. TFG-beta on page 4?
3. F9 gene to be italicized (page 5, Results).
4. Top of page 14 "bypassing agents" not "bypass reagent".
5. Bray reference "American Journal of Hematology"
6. Hausl reference "Thrombosis and Haemostasis" Likewise Metzner reference.
7. Jadhav and Warrier reference "Seminars in Thrombosis and Haemostasis"
8. Martino et al reference is incomplete (volume, pages omitted). Likewise McIntosh reference.
9. Nathwani et al reference "New England Journal of Medicine".
10. Nayak reference "Frontiers in Microbiology". Likewise Simioni et al reference.
11. Wang et al reference "Gene Therapy".

1st Revision - authors' response 18 July 2013

We would like to thank the reviewers for their support, constructive criticism, and recognition that this manuscript is timely and of great relevance for translational medicine.

Referee#1:

The authors address the problem of inhibitor-positive haemophilia in a gene therapy mouse model. The authors conclude that AAV-mediated gene therapy has a tolerisation effect through induction of specific Treg cells. These experiments are well-performed, and convincingly demonstrate induction of specific Treg and depletion of memory B cells in vivo. These data are largely observational however, and the immunological mechanism of Treg induction and memory B cell depletion is not elucidated.

1. *To what extent does leaky transduction of other non-hepatic cell types (and cross presentation) contribute to the tolerization process.*

It is well documented in the literature that AAV serotype 8 fails to yield any detectable transgene expression in professional antigen presenting cells *in vivo* and also fails to transduce dendritic cells *in vitro*, even if a non-specific promoter is used (Lu & Song, 2009; Vandendriessche et al, 2007). This is now explained in the manuscript. The ApoE/hAAT enhancer/promoter used in our study has been shown to drive liver specific expression of transgenes in multiple different vector systems (Miao et al, 2000; Okuyama et al, 1996) and was further characterized for specificity in investigational new drug (IND) applications for use in clinical trials for hepatic AAV2 and AAV8 gene therapy for haemophilia (Children's Hospital of Philadelphia, St. Jude's Children's Research Hospital/University College London). Consistent with published data by Nathwani and colleagues (Nathwani et al, 2006), Figure 5, we find FIX transgene expression from an AAV8-ApoeE/hAAT-FIX vector undetectable by quantitative RT-PCR in non-hepatic tissues (despite biodistribution of vector genomes to these tissues, albeit at lower levels than to the liver). Immunostaining confirmed lack of FIX expression in the spleen and in resident macrophages in the liver. Only cells showing size and morphology of hepatocytes (but not sinusoidal cells) showed FIX expression. Both sets of data have now been added to the manuscript in the form of supporting information **Figure S1**.

Nonetheless, the reviewer is making two excellent points with regard to extrahepatic transgene expression and the role of antigen presentation. Work by us with AAV vectors and by the Naldini lab (using microRNA regulated LVs) has established that hepatocyte-restricted expression is an excellent means of tolerance induction. In contrast, gene transfer to a number of other tissues is often immunogenic. However, robust hepatic transgene expression induces a dominant state of tolerance that suppresses responses in other tissues. Hence, successive (or even simultaneous) hepatic - extrahepatic gene transfer (to skeletal and cardiac muscles, CNS, and others) has been a successful strategy for tolerance induction, while immune responses were observed if the hepatic gene transfer arm was not included. This is now explained in the revised manuscript. For diseases such as lysosomal storage disorders, affecting multiple organs, this strategy is considered for future clinical trials see (Hoffman et al, 2007; Logan & Alexander, 2012; Passini et al, 2007; Zhang et al, 2012).

While transgene expression in hepatocytes induces tolerance, the question arises whether a professional antigen presenting cell (APC) is required to cross-present hepatocyte-derived antigen to $CD4^+$ T cells. As shown very elegantly by Brown and Naldini, direct transgene expression in bone marrow-derived APCs is detrimental to tolerance induction(Brown et al, 2006). To our advantage, many AAV serotypes such as AAV8 are naturally inefficient in transducing professional APCs, and proper choice of the promoter can robustly drive transgene expression in hepatocytes. Non-APCs are not expected to express MHC II molecules, although some literature suggests that hepatocytes may express a low level of MHC II. To try to address a requirement for APCs, we have conducted new studies, the results of which are now included as supplementary **Figure S4**. In order to be able to track transgene product-specific $CD4^+$ T cells, we conducted ovalbumin gene transfer to BALB/c mice using our previously published AAV8-ova vector, which directs robust expression of secreted ova in hepatocytes(Cooper et al, 2009; Dobrzynski et al, 2004). Fluorescently labelled $CD4^+$ T cells from BALB/c mice transgenic for the ova-specific DO11.10 T-cell receptor (TCR) readily proliferated *in vivo* after

transfer to AAV-ova transduced mice. In contrast, ova-specific T cell proliferation was substantially reduced or even completely eliminated upon Kupffer cells/M1 macrophage inactivation or depletion of $CD11c^+$ DCs. Therefore, MHC II-restricted presentation of transgene product-derived antigen induces proliferation of $CD4^+$ T cells, a process that requires professional APCs and thus cannot be explained by direct antigen presentation by hepatocytes. These data do not entirely prove but at least support a model of professional APCs playing an intermediary role in cross presentation of hepatocyte-derived antigen to $CD4^+$ T cells.

Our main finding is that hepatic gene transfer with an AAV vector at levels of transgene expression currently achieved in clinical trials effectively reverses FIX inhibitors and desensitizes haemophilic animals even in the face of anaphylaxis. As pointed out by reviewers 2 and 3, these data are of high relevance for translational medicine and should therefore be ideally suited for a journal with such a focus. Nonetheless, the manuscript is not merely observational and contains a number of mechanistic data that help explain how tolerance was established (vector dose-dependent induction of $CD4^+CD25^+$ Treg, continuous requirement for active suppression by Treg, antigen dose-dependent suppression of memory B cells, suppression of T help, and the ability of recovering Treg to re-establish tolerance). More mechanistic data have now been added with regard to antigen presentation. We agree that the mechanism of Treg generation in response to hepatic antigen and of memory B cells by high-dose antigen are important basic immunological questions, which we are pursuing but are beyond the scope of this manuscript.

2. *The length of the follow up experiments is limited. It would be important to show durability over extended periods.*

Please note that time lines in the graphs in figure 2 begin with the time of gene transfer, i.e. ~2 months after first injection of FIX. We followed the haemophilic mice for up to ~5 months post gene transfer, approximately 7 months since the first exposure to FIX protein. At the time of sacrifice, the mice were approximately 9 months old, ~50% of the life span of a male C3H/HeJ mice. Once this particular strain approaches 1 year of age, several health problems arise unrelated to haemophilia. These include occurrences of prolapsed penis, opportunistic bacterial infections of the lung, and complications from loss of tail tissue as a result of repeated tail bleeds/cuts required to monitor inhibitor formation. Older C3H/HeJ mice also have a high incidence of hepatomas (70-90% by 14 months of age). It would be difficult to follow these animals much longer than what we already performed, as our animal care services typically request euthanasia as more severe health problems arise. Further, studies of immune responses are not optimal in aged mice.
At the same time, we have shown that the tolerance mechanism is very robust. Even when we completely eliminated Treg in the conditional FoxP3 knockouts, tolerance was re-established as soon as Treg recovered. Also, FIX expression from hepatic AAV gene transfer is known to be stable for the lifetime of a mouse. Tolerized hemophilic mice had no detectable B or effector T cell response to FIX at the time of sacrifice (~9 months of age). All these factors make it highly unlikely that a response would have re-emerged in the limited additional time we may have been able to further follow these mice. Such long-term experiments could be done in a more meaningful way in the haemophilia B dog models. Nonetheless, given

© EMBO

that the haemophilia B C3H/HeJ model is currently the only one that mimics inhibitor formation and anaphylaxis to FIX in response to intravenous replacement therapy (as performed in humans), and given the on-going experience with sustained FIX expression in the therapeutic range with AAV-FIX in current Phase I/II clinical trials, we would argue that the data contained in this manuscript directly support clinical experimentation with this approach to treat FIX inhibitors. The notion of high relevance of these data for medical treatment is further supported by the other reviewers.

Referee #2

This is an interesting study showing that AAV8 vector mediated hFIX expression can reverse pre-existing anti-hFIX antibody responses in mice and thereby eliminate anaphalaxis and neutralizing antibody responses. The mechanism is complex and is shown to be due to induction of Tregs that suppress B cell memory responses and antibody production. It is notable that AAV-mediated suppression of FIX antibodies was possible at FIX levels of about 6% of normal and therefore potentially achievable in human gene therapy trials. Therefore, this work is medically important because it suggests that recently described AAV gene therapy directed at the liver can now be extended to patients with pre-existing neutralizing antibodies. The experiments are well designed and properly controlled and interpreted. The manuscript is clearly written and organized.

Per request by the editor we have added into the discussion speculation on why different levels of hFIX protein (6% versus 100%) were required for inhibitor reversal in our study as compared to a companion manuscript using a liver directed *hF9* lentiviral vector.

On the one hand, our model more closely mimics the immune response in humans as C3H/HeJ F9-/- mice develop inhibitors and anaphylactic reactions upon repeated intravenous delivery of FIX concentrate (in a F9 gene deletion setting). It is indeed encouraging that levels of FIX expression already achieved in several patients in the clinic with AAV8-FIX gene transfer are sufficient to reverse inhibitors in this model. On the other hand, it is possible that the LV gene transfer described in the companion manuscript may have also been effective at lower doses in our model. Because of the different mouse strain, an adjuvant was required to induce the antibody response to FIX, which however may in the end have further heightened the immune response to a degree where a high dose regimen was needed.

Referee #3

Markusic et al convincingly show that they can tolerize previously immunized mice with established anti-factor IX antibodies with liver-directed gene transfer using AAV vectors in haemophilia B mice. This is an important problem in humans for various reasons, particularly that factor IX inhibitors prevent proper therapy for bleeding and/or prophylaxis against bleeding, and inhibitors to factor IX can be associated with anaphylaxis and/or nephrotic syndrome in the event of repeat exposure to factor IX. In fact, the anaphylaxis in humans was first noted in patients undergoing standard desensitization with human factor IX proteins. Thus, a safe, effective approach to treating these inhibitors would be welcome.

Issues/Comments/Concerns

1. *Citation for 1 in 20,000 male births statistic in introduction? More commonly see 1:30,000 elsewhere.*

We thank the referee for pointing this out and we have corrected the text to read 1:30,000 as commonly reported by others.

2. *May wish to state in the introduction that inhibitors occur predominantly in patients with severe hemophilia.*

We agree and have added a line in the introduction that states that inhibitors predominantly form in patients with severe haemophilia.

3. *May wish to mention in introduction nephrotic syndrome with factor IX ITI (Ewenstein et al, Blood. 1997;89:1115-1116) and also in Discussion on page 12.*

We agree that this is an important paper describing the development of nephrotic syndrome in multiple patients following hFIX immune tolerance induction. It was not initially included as it was referenced and mentioned in the cited reviews. We have now included this reference within the introduction and discussion as requested.

4. *Would be curious to know if this C3H/HeJ background strain is one that is immune to toll-receptor mediated septic shock with exposure to endotoxin? See discussion of C3H strain on page 6.*

Indeed, the C3H/HeJ strain is deficient for toll like receptor 4 (TLR4), which senses the endotoxin lipopolysaccharide (LPS). Hence, this strain does not experience toxicity from LPS administration. We are currently investigating the impact of this on immune responses against hFIX protein by crossing C3H/HeJ $F9^{-/-}$ females with congenic C3H/OuJ males, which have a functional TLR4. It is theoretically possible that the mutation in TLR4 increases susceptibility to environmental factors that may increase the risk of inhibitor formation. However, these studies are only in early stages.

5. *With regard to mild IgG response to factor IX in Balb/c mice (page 6), this has been shown previously by Lozier et al 2005;105:1029-1035 with strong adjuvants (adenovirus). Likewise for mention of low reactivity of C57BL/6 mice on page 10.*

We would like to thank the referee for pointing out this paper. Upon review it does indeed show reduced IgG in C57BL/6 and Balb/c mice compared with C3H following injection with an adenovirus expressing hFIX protein. We have included this paper as a reference in regards to low IgG reactivity against hFIX protein in Balb/c and C57BL/6 mice.

6. *Materials and Methods section, page 19. Definition of Bethesda unit is awkward/incomplete. One BIAU is the reciprocal of the dilution of plasma that neutralizes 50% of the factor IX in a normal plasma sample with which it is mixed in vitro.*

We would like to thank the referee for pointing this out and have amended the definition to reflect what the referee has provided.

7. *Figure 1 is unreadable as presented, if my printout is any guide, it needs to be at least twice as large to be readable, and cannot fit into a single column as attempted here. Other figures are satisfactory; in fact, Figure 5 could be made slightly smaller and not suffer for the effort.*

The initial layout of figure 1 was 3 x 2 panels and we had anticipated that this would cover the whole width of the page. We have remade figure 1 to be 2 x 3 panels and have increased the font size to hopefully make the figure more readable. We will look into reducing the size of Figure 5.

Minor issues:

1. Factor IX is abbreviated "FIX" in most journals, rather than F.IX.

We have changed the abbreviation of Factor IX to FIX.

2. TFG-beta on page 4?

We have corrected this to TGF-beta.

3. F9 gene to be italicized (page 5, Results)

We have gone through the manuscript and italicized all mention of the F9 gene including the instance mentioned above on page 5, Results.

4. Top of page 14 "bypassing agents" not "bypass reagent".

We have changed the text to read bypassing agents.

5-11. We have made changes to the references as requested.

References

Brown BD, Venneri MA, Zingale A, Sergi Sergi L, Naldini L (2006) Endogenous microRNA regulation suppresses transgene expression in hematopoietic lineages and enables stable gene transfer. Nat Med 12**:** 585-591

Cooper M, Nayak S, Hoffman BE, Terhorst C, Cao O, Herzog RW (2009) Improved induction of immune tolerance to factor IX by hepatic AAV-8 gene transfer. Hum Gene Ther 20**:** 767-776

Dobrzynski E, Mingozzi F, Liu YL, Bendo E, Cao O, Wang L, Herzog RW (2004) Induction of antigen-specific CD4+ T-cell anergy and deletion by in vivo viral gene transfer. Blood 104**:** 969-977

Hoffman BE, Dobrzynski E, Wang L, Hirao L, Mingozzi F, Cao O, Herzog RW (2007) Muscle as a target for supplementary factor IX gene transfer. Hum Gene Ther 18**:** 603-613

Logan GJ, Alexander IE (2012) Adeno-associated virus vectors: immunobiology and potential use for immune modulation. Curr Gene Ther 12**:** 333-343

Lu Y, Song S (2009) Distinct immune responses to transgene products from rAAV1 and rAAV8 vectors. Proc Natl Acad Sci U S A 106**:** 17158-17162

Miao CH, Ohashi K, Patijn GA, Meuse L, Ye X, Thompson AR, Kay MA (2000) Inclusion of the hepatic locus control region, an intron, and untranslated region increases and stabilizes hepatic factor IX gene expression in vivo but not in vitro. Mol Ther 1**:** 522-532

Nathwani AC, Gray JT, Ng CY, Zhou J, Spence Y, Waddington SN, Tuddenham EG, Kemball-Cook G, McIntosh J, Boon-Spijker M et al (2006) Self-complementary adeno-associated virus vectors containing a novel liver-specific

human factor IX expression cassette enable highly efficient transduction of murine and nonhuman primate liver. Blood 107**:** 2653-2661

Okuyama T, Huber RM, Bowling W, Pearline R, Kennedy SC, Flye MW, Ponder KP (1996) Liver-directed gene therapy: a retroviral vector with a complete LTR and the ApoE enhancer-alpha 1-antitrypsin promoter dramatically increases expression of human alpha 1-antitrypsin in vivo. Hum Gene Ther 7**:** 637-645

Passini MA, Bu J, Fidler JA, Ziegler RJ, Foley JW, Dodge JC, Yang WW, Clarke J, Taksir TV, Griffiths DA et al (2007) Combination brain and systemic injections of AAV provide maximal functional and survival benefits in the Niemann-Pick mouse. Proc Natl Acad Sci U S A 104**:** 9505-9510

Vandendriessche T, Thorrez L, Acosta-Sanchez A, Petrus I, Wang L, Ma L, L DEW, Iwasaki Y, Gillijns V, Wilson JM et al (2007) Efficacy and safety of adeno-associated viral vectors based on serotype 8 and 9 vs. lentiviral vectors for hemophilia B gene therapy. J Thromb Haemost 5**:** 16-24

Zhang P, Sun B, Osada T, Rodriguiz R, Yang XY, Luo X, Kemper AR, Clay TM, Koeberl DD (2012) Immunodominant liver-specific expression suppresses transgene-directed immune responses in murine pompe disease. Hum Gene Ther 23**:** 460-472

2nd Editorial Decision 08 August 2013

Thank you for the submission of your revised manuscript to EMBO Molecular Medicine.

As mentioned in an earlier letter, we have experienced a delay due to the fact that Reviewer 1, whom we asked to re-evaluate your revised manuscript, is on vacation. We therefore asked one of the other Reviewers (#3) to check whether you had adequately replied to this Reviewer's criticisms. As you will see the s/he is satisfied and is globally supportive.

I am pleased to inform you that we will be able to accept your manuscript pending the following final amendments:

- As per our Author Guidelines, the description of all reported data that includes statistical testing must state the name of the statistical test used to generate error bars and P values, the number (n) of independent experiments underlying each data point (not replicate measures of one sample), and the actual P value for each test (not merely 'significant' or 'P < 0.05').

Please provide a final version of your manuscript without the coloured lettering (no longer needed).

Please submit your revised manuscript within two weeks. I look forward to seeing a revised form of your manuscript as soon as possible.

I look forward to reading a new revised version of your manuscript as soon as possible.

***** Reviewer's comments *****

To the best of my ability to comprehend, both sets of Authors have replied to Reviewer 1's criticisms in a fully satisfactory way that adds value to each paper.

2nd Revision - authors' response 15 August 2013

As per our Author Guidelines, the description of all reported data that includes statistical testing must state the name of the statistical test used to generate error bars and P values, the number (n) of independent experiments underlying each data point (not replicate measures of one sample), and the actual P value for each test (not merely 'significant' or 'P < 0.05').

Please provide a final version of your manuscript without the coloured lettering (no longer needed).

Response:

The further revised version of this manuscript now includes clarification of all statistical tests used, the number of independent experiments/animals underlying each data point, and the actual *P* values for each test which are now reported in the appropriate figure legends. This style of reporting was selected based on review of several recently published manuscripts in EMBO Molecular Medicine. We have carefully reviewed the revised manuscript and have removed all blue text as requested.
