# Supplementary material for: Effective gene therapy for haemophilic mice with pathogenic factor IX antibodies
Source: EMBO Mol Med. 2013 Sep 16;5(11):1698–709. doi: 10.1002/emmm.201302859 (PMC3840486; doi:10.1002/emmm.201302859)
Supplement: Supplementary file 2 [file emmm0005-1698-SD2.pdf]

Effective Gene Therapy for Hemophilic Mice with Pathogenic Factor IX Antibodies

David M. Markusic[1], Brad E. Hoffman[1], George Q. Perrin[1], Sushrusha Nayak[1], Xiaomei Wang[1], Paul A. LoDuca[1], Katherine A. High[2,3,4], Roland W. Herzog[1]

[1]Department of Pediatrics, University of Florida, Gainesville, FL [2]Department of Pediatrics, The Children's Hospital of Philadelphia and University of Pennsylvania Medical Center, Philadelphia, PA [3]Center for Cellular and Molecular Therapeutics, The Children's Hospital of Philadelphia, Philadelphia, PA [4]Howard Hughes Medical Institute, Philadelphia, PA

Supporting information

Figure S1. AAV8-ApoEe/hAAT-*hF9* bio-distribution and liver specific expression of hFIX protein....... 2
Figure S2. Recovery of Treg following two doses of DT. 3
Figure S3. Residual hF.IX ASC detected in a "late DT" mouse with delayed Treg reconstitution....... 4
Figure S4. Requirement for professional antigen presenting cells (APCs) for transgene product-specific CD4$^+$ T cell proliferation. 5
Supporting methods: 6

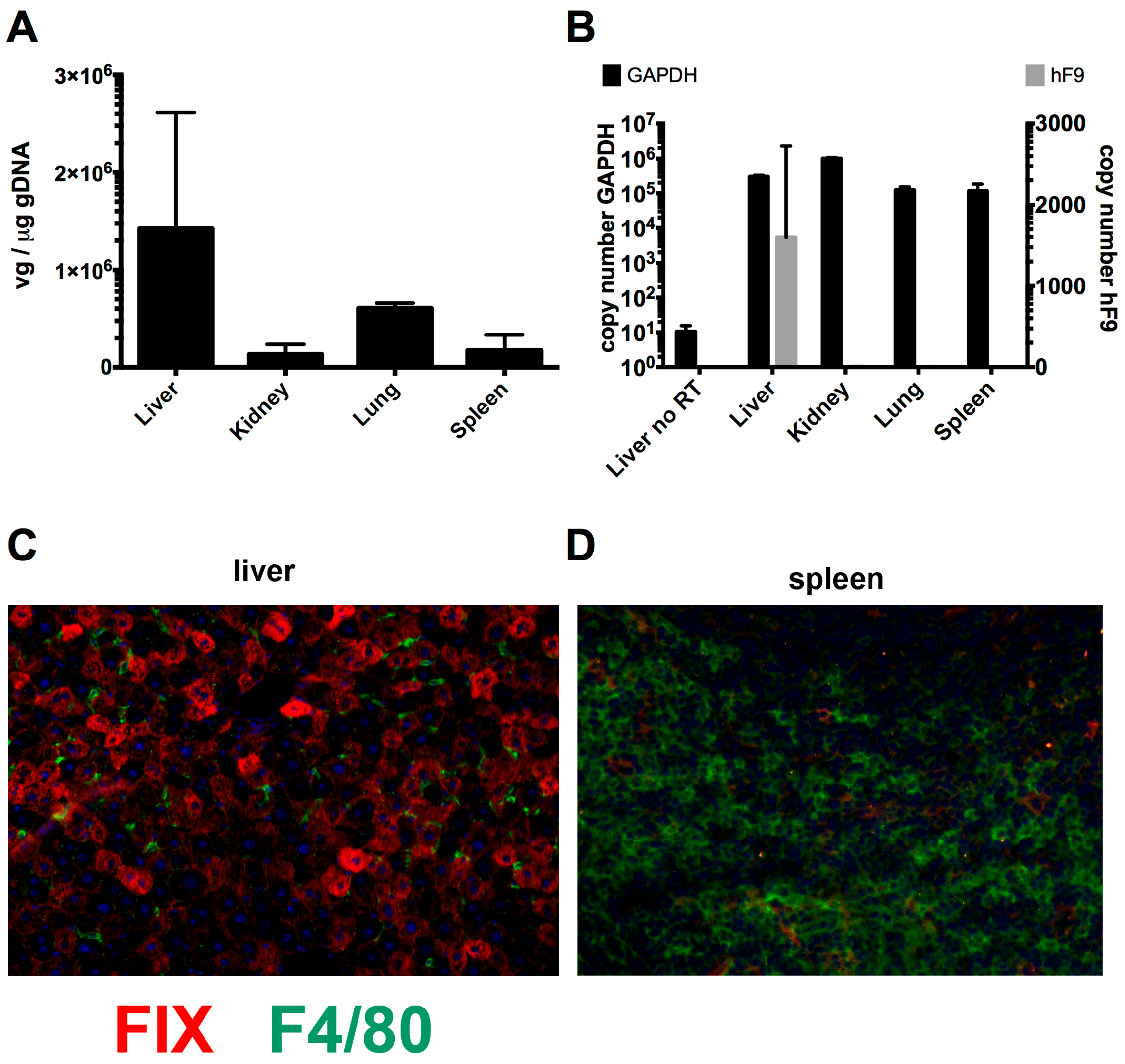

Figure S1. AAV8-ApoEe/hAAT-*hF9* bio-distribution and liver specific expression of hFIX protein.

Vector was given by tail vein injection at 1x10^{11} vg/mouse to hemophilia B mice (C3H/HeJ F$9^{-/-}$). (A) AAV8-ApoEe/hAAT-*hF9* bio-distribution performed on genomic DNA isolated from liver, kidney, lung, and spleen three weeks following IV administration in C3H/HeJ *F9*$^{-/-}$ mice (n=3). (B) *hF9* mRNA expression determined by RT-qPCR performed on total RNA isolated from liver, kidney, lung, and spleen three weeks following IV administration in C3H/HeJ *F9*$^{-/-}$ mice (n=3). *GAPDH* copy numbers in black (left axis) and *hF9* copy numbers in grey (right axis). A no reverse transcriptase control was included for liver RNA samples (n=3). (C) Representative liver section stained for hFIX (red) and F4/80 Kupffer cells and macrophages (green). (D) Representative spleen section stained for hFIX (red) and F4/80 macrophages (green).

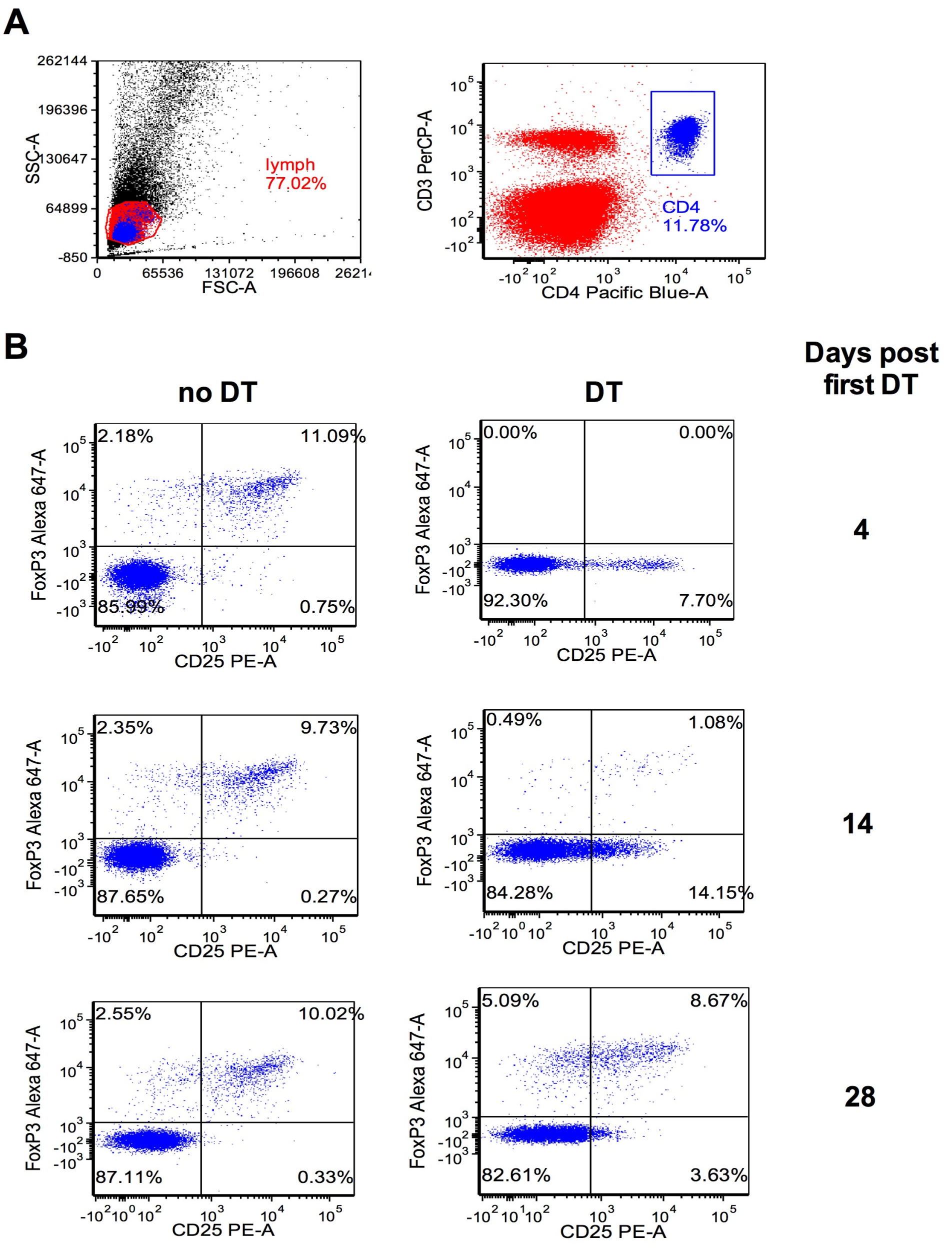

Figure S2. Recovery of Treg following two doses of DT.
(A) Gating scheme of mouse PBMC using CD3-PerCP-CY5.5 and CD4-e450 gates to select CD4$^+$ T cells. (B) Representative dot plots used to calculate %CD25$^+$ FoxP3$^+$ Treg for control untreated DTR-FoxP3 mice (left column) and DT treated DTR-FoxP3 mice (right column) with day 0 as the first DT injection.

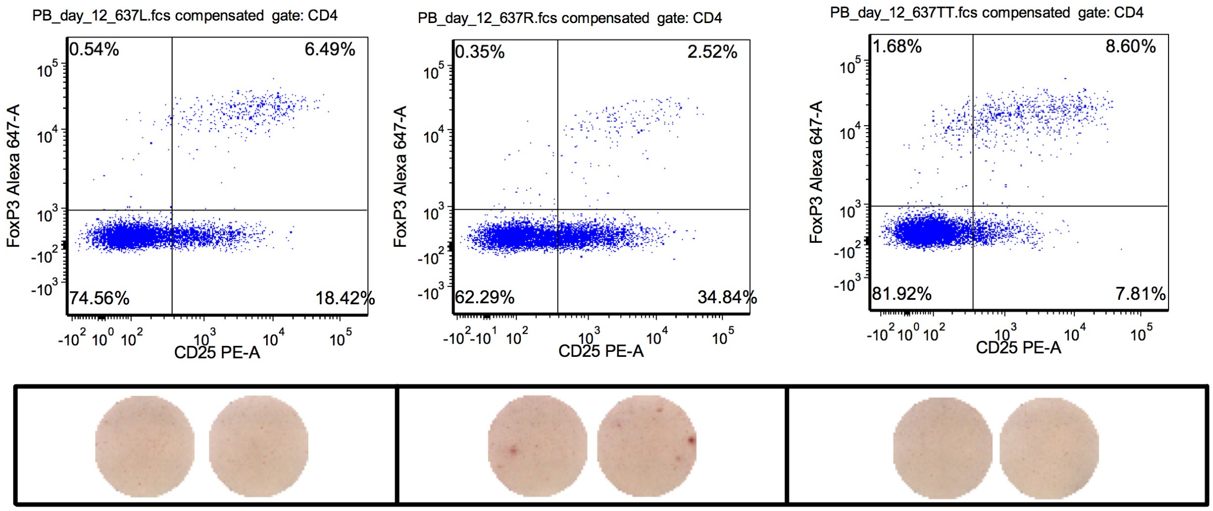

Figure S3. Residual hF.IX ASC detected in a "late DT" mouse with delayed Treg reconstitution.
(A) Flow cytometry staining of peripheral blood 14 days following late DT treatment. (B) hF.IX B cell ELISpot on splenocytes from late DT treated mice isolated 29 days post DT treatment.

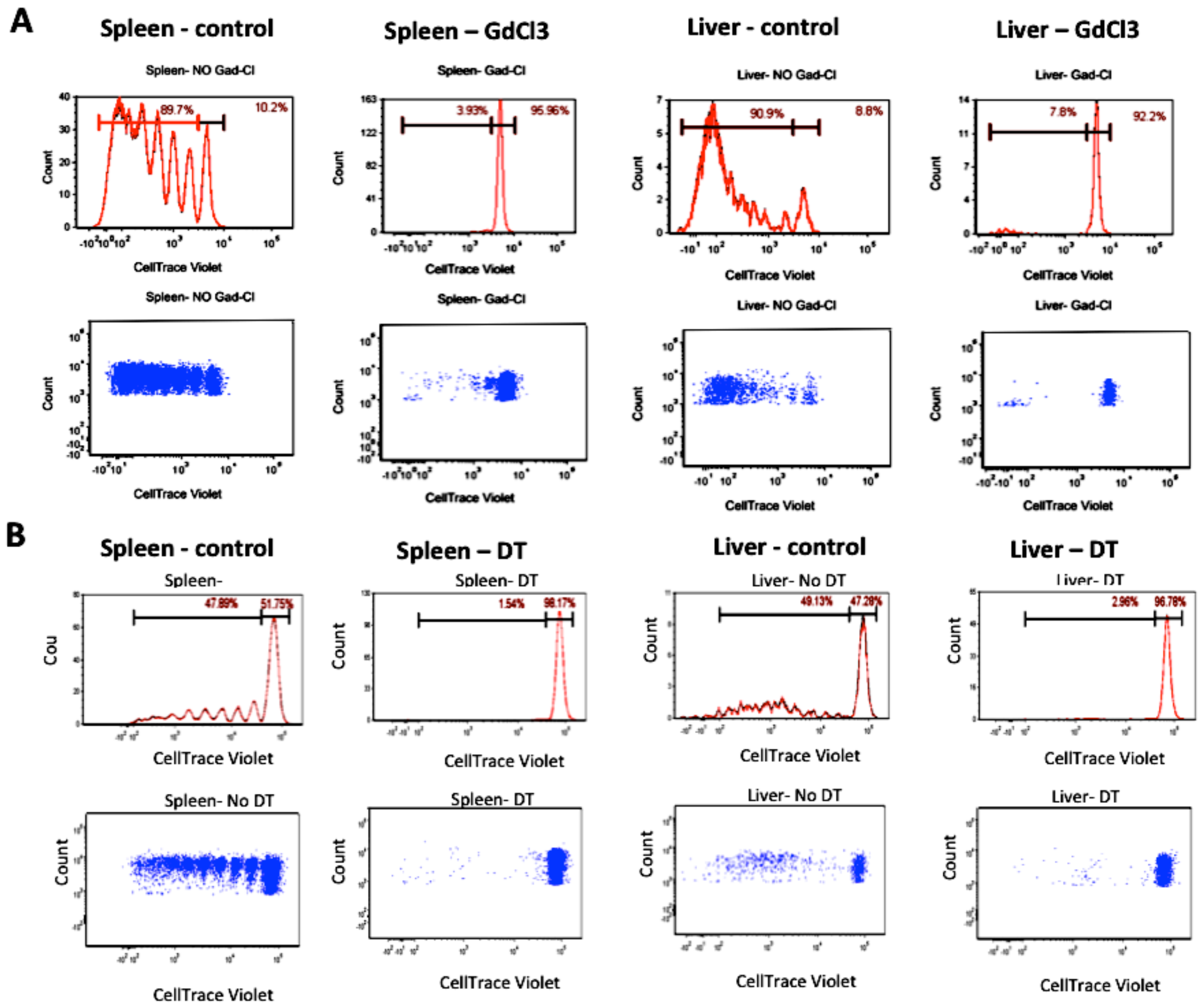

Figure S4. Requirement for professional antigen presenting cells (APCs) for transgene product-specific CD4$^+$ T cell proliferation.

AAV8-EF1α-ova vector was administered by tail vein injection to BALB/c mice at $1x10^{11}$ vg/mouse. (A) Proliferation of transferred T cells in spleens and livers of BALB/c mice. Left panel: control animal. Right panel: animal, in which Kupffer cells and M1 macrophages were inactivated using gadolinium chloride ($GdCl_3$). (B) Proliferation of transferred T cells in spleens and livers of conditional CD11c deficient mice (CD11-DTR BALB/c mice). Left panel: control animal. Right panel: animal, in which CD11c$^+$ DCs cells were depleted following administration of diphtheria toxin (DT). Shown are representative examples for n=3 per experimental group. Percent cells that have or have not proliferated are indicated. No proliferation was observed in BALB/c mice that had not received AAV-ova vector (data not shown).

Supporting methods:

Vector genome bio-distribution
C3H/HeJ *F9*$^{-/-}$ mice were injected in the tail vein with $1x10^{11}$ vg of AAV8-*hF9* vector. Three weeks post-injection tissues (liver, kidney, lung, and spleen) were collected and processed the same day. Tissue (liver, kidney, lung and tissue) for DNA extraction were placed into PBS and processed using a Qiagen DNEasy Blood and Tissue extraction kit per manufacturer's instructions. rAAV vector genome copies in liver, kidney, heart, lung and spleen gDNA samples were quantified by real time PCR using an ABI 7900 HT sequence detection system (Applied Biosystems, Carlsbad, CA, USA) according to the manufacture's instructions and results were analyzed using the SDS 2.3 software. Briefly, primers and probe were designed to the hAAT promoter region of the rAAV vector. A standard curve was prepared using plasmid DNA containing the same hAAT promoter sequence. PCR reactions contained a total volume of 100µL and run under the following conditions: 50°C for 2 minutes, 95°C for 10 minutes, and 45 cycles of 95°C for 15 seconds and 60°C for one minute. DNA samples were assayed in triplicate. In order to asses PCR inhibition, the third replicate was spiked with plasmid DNA at a ratio of 100 copies/µg gDNA. If this replicate was greater than 40 copies/µg gDNA then the results were considered acceptable. If a sample contained greater than or equal to 100 copies/µg gDNA, the sample was considered positive for vector genomes. If a sample contained less than 100 copies/µg gDNA it was considered negative for vector genomes. The vector copy number reported was normalized per 1.0 µg gDNA.

hAAT Forward
GTAGGCGGGCGACTCAGA
hAAT Reverse
CCCAGTTATCGGAGGAGCAA
hAAT Probe
6FAM-CCCAGCCAGTGGACTTAGCCCCTG

RT-qPCR for *hF9* mRNA expression
Tissue for RNA extraction was cut into small pieces and immediately placed in RNA later solution and RNA was extracted using Qiagen RNAEasy kit per manufacturer's instructions. Approximately 1 ug of total RNA per tissue was used to generate cDNA using SABiosciences RT^2 First Strand synthesis kit per manufacturer's instructions. Primers specific for murine *GAPDH* (PPM02946E) and human *F9* (PPH07069B) were purchased from SABiosciences and RT-qPCR was performed on a Bio-Rad MyiQ instrument using RT^2 qPCR mastermix from SABiosciences all according to manufacturer's instructions. Plasmid standards for murine *GAPDH* and human *F9* were used to determine relative copy numbers in the different tissue samples. A no reverse transcriptase control for liver RNA was used to check for any contamination of genomic DNA or vector genomes.

Immunostaining of liver and spleen for hFIX expression
Liver and spleen were cut into approximately 1cm pieces and frozen in liquid nitrogen using Tissue-Tek cryomolds (4557) and Optimal Cutting Temperature (OCT) Compound. Seven micron tissue sections were mounted on poly-lysine coated slides and tissue was stained as previously described. Briefly tissue sections were brought to room temperature and fixed in fresh acetone for 10 minutes, removed and allowed to air dry. Following several washes in PBS tissues were blocked in PBS containing 5% Donkey serum and incubated with primary antibodies (1:200 Goat anti human FIX and 1:100 Rat anti mouse F4/80) for 30 minutes. Following several washes in PBS tissues were then incubated with secondary antibodies (1:200 Donkey anti Goat Alexaflour-598 and 1:200 Donkey anti Rat alexaflour-488) for 30 minutes.

Slides were washed in PBS, rinsed in water, and mounted with ProLong Gold with DAPI. Images were captured on a Nikon Eclipse 80i using a Retiga 2000R camera and NIS-Elements software.

***In vivo* T cell proliferation.**
Male 6–8-week-old BALB/c mice were purchased from Jackson Laboratories (Bar Harbor, ME) and used for Kupffer cell inactivation experiments. Mice were injected via tail vein with 10 mg/kg of gadolinium chloride (GdCl3) two weeks after AAV8-EF1α-ova vector administration and 48 and 24 hours prior to adoptive transfer of DO11 T cells, as previously described (Zaiss *et al*., 2002). For CD11c dendritic cell depletion experiments, male 6-8-week old C.FVB-Tg(Itgax-DTR/EGFP)57Lan/J mice on the BALB/c background (CD11-DTR BALB/c) were purchased from Jackson Laboratories. These mice carry the receptor for DT under control of the CD11c promoter so that treatment with DT results in transient depletion of CD11c+ dendritic cells. These mice were intraperitonealy injected with 4 ng/g of DT (LIST Biologics, Campbell, CA) diluted in 200 µl PBS, as described previously (Jung et al., 2002). For T cell proliferation experiments, male DO11.10 TCR transgenic mice (BALB/c background, 6-8 weeks of age) were originally purchased from Jackson Laboratories and bred at the University of Florida's Animal Laboratory Facilities. All studies were in accordance with protocols approved by Institutional Animal Care and Use Committees at the University of Florida, Gainesville.
CD4+ T cells were isolated from spleens of donor BALB/c-DO11 mice using the Miltenyi CD4 Isolation Kit II (Auburn, CA). Cells were then labeled with 3 µmol/l CellTrace Violet Cell Proliferation Kit (Invitrogen, Carlsbad, CA) per the manufacturers instructions. Five to ten million labeled cells were then adoptively transferred to recipient mice in 200µl PBS by tail vein injection. At 3-4 days after adoptive transfer, splenocytes or liver lymphocytes of recipient mice were harvested. Spleens were disrupted with a 70µ cell strainer and subjected to RBC lysis prior to staining for flow cytometry. Liver lymphocytes were isolated by mincing the liver and disrupting tissue through a 70µ cell strainer. Hepatocytes were removed by centrifuging at 30g for 3 minutes. Supernatant cells were then pelleted, loaded on a 30% Histodenz (Sigma, St. Louis, MO) gradient and centrifuged at 1500g for 20 minutes. Hepatic lymphocytes were subjected to RBC lysis prior to staining for flow cytometry. Lymphocytes were stained with antibodies to CD4 and DO11-TCR. They were then analyzed by flow cytometry. Gates were set to identify DO11 CD4+ T cells of donor origin. These cells were then analyzed for decreases in CellTrace Violet expression, with a decrease being indicative for cell proliferation. The percentage of proliferating cells was calculated by gating on the populations displaying a decrease in CellTrace Violet intensity, as compared with the non-proliferating cells displaying the highest CellTrace Violet intensity.

References:

Zaiss AK, Liu Q, Bowen GP, Wong NC, Bartlett JS, Muruve DA. Differential activation of innate immune responses by adenovirus and adeno-associated virus vectors. J Virol. 2002;76:4580-4590.

Jung S, Unutmaz D, Wong P, Sano G, De los Santos K, Sparwasser T, Wu S, Vuthoori S, Ko K, Zavala F, Pamer EG, Littman DR, Lang RA. In vivo depletion of CD11c+ dendritic cells abrogates priming of CD8+ T cells by exogenous cell-associated antigens. Immunity. 2002 Aug;17(2):211-20.
